# Next frontier in tumor immunotherapy: macrophage-mediated immune evasion

**DOI:** 10.1186/s40364-021-00327-3

**Published:** 2021-10-09

**Authors:** Yingqi Qiu, Tong Chen, Rong Hu, Ruiyi Zhu, Chujun Li, Yingchen Ruan, Xiaoling Xie, Yuhua Li

**Affiliations:** 1 Department of Hematology, Zhujiang Hospital, Southern Medical University, No. 253 GongyeDadaoZhong, Guangzhou, Guangdong 510280 P. R. China; 2 The Second School of Clinical Medicine, Southern Medical University, No. 1838 GuangzhongDadaoBei, Guangzhou, Guangdong 510515 P. R. China; 3 Shunde Hospital, Southern Medical University (The First People’s Hospital of Shunde Foshan), Foshan, 528308 China; 4 Bioland Laboratory (Guangzhou Regenerative Medicine and Health Guangdong Laboratory), Guangzhou, 510005 P. R. China

**Keywords:** Tumor-associated macrophages, Immune evasion, Tumor microenvironment

## Abstract

Tumor-associated macrophages (TAMs), at the core of immunosuppressive cells and cytokines networks, play a crucial role in tumor immune evasion. Increasing evidences suggest that potential mechanisms of macrophage-mediated tumor immune escape imply interpretation and breakthrough to bottleneck of current tumor immunotherapy. Therefore, it is pivotal to understand the interactions between macrophages and other immune cells and factors for enhancing existing anti-cancer treatments. In this review, we focus on the specific signaling pathways through which TAMs involve in tumor antigen recognition disorders, recruitment and function of immunosuppressive cells, secretion of immunosuppressive cytokines, crosstalk with immune checkpoints and formation of immune privileged sites. Furthermore, we summarize correlative pre-clinical and clinical studies to provide new ideas for immunotherapy. From our perspective, macrophage-targeted therapy is expected to be the next frontier of cancer immunotherapy.

## Introduction

Despite promising prospects, most immunotherapies have encountered bottlenecks in response rate, toxicity and drug resistance at present [1]. This may be attributed to the fact that the crosstalk between tumor cells and various cells in the Tumor microenvironment (TME) inhibits immune surveillance mediated by immune cells, inducing tumor immune escape and tumor progression. Therefore, more researches should be done about immune escape in order to surmount the resistance to existing therapies caused by TME. Host immune system dysfunction, such as T cell anergy, excessive existing of regulatory T cells (Tregs), is one of the main mechanisms of tumor escaping from immune surveillance. In addition, tumor related factors, including secretion of immunosuppressive cytokines, resistance to apoptosis and antigen deletion, may also associate with immune escape [2]. Host and tumor related mechanisms could lead to the failure of establishing appropriate anti-tumor specific immune response, which are usually the key factors limiting the success of cancer immunotherapy.

Macrophages, which significantly influence anti-infection immunity and homeostasis of internal environment by mediating innate immunity and helping to start adaptive immunity, have been demonstrated to be essential in immune escape by mechanisms described above [3]. They are differentiated from circulating monocytes stimulated by granulocyte macrophage colony-stimulating factor (GM-CSF) or M-CSF, which originate from bone marrow-derived progenitor cells [4]. In the light of the makeup of the cytokine milieu and the surrounding tissular niche, they can differentiate into a wide range of phenotypic states, which may change as a spectrum or an orthogonal cross, or even in accordance with a snow-like multiple-axis and multiple-branch pattern [5]. Although the range of macrophages activation status is complex, it is generally simplified into two categories: classically activated macrophages (M1) and alternatively activated macrophages (M2) respectively. Exposed to factors such as IFN-γ, TNF-α and lipopolysaccharide, macrophages can polarize into M1 characterized by the expression of CD68, CD86 and CD80, which secrete cytokines and chemokines like TNF-α, IL-1β, IL-12, CXCL9, CXCL10, to promote the pro-inflammatory Th1 response. M2 with high expression of CD163, CD204 and CD206 are induced by IL-4 and IL13, which exert immunomodulatory effects and plays a key role in inhibiting endogenous antitumor immunity [6]. Significantly, TAMs, mainly referring to M2, have been identified to secrete inhibitory cytokines and affect immune cells, creating a favorable immunosuppressive TME for tumor progression and immunotherapy resistance [7, 8]. The recent advent of technologies single-cell RNA sequencing (scRNA-seq) is one approach to dissect the heterogeneity of complex biological systems. Based on clinical tumor samples or corresponding mouse models of tumor tissue, analyses of various tumors such as breast cancer and atypical teratoid/rhabdoid tumors (ATRT) utilizing scRNA-seq and time-of-flight mass cytometry (CyTOF) have proved that M2 is the major infiltrating cell in tumor tissues, which overexpress angiogenesis or epithelial-mesenchymal transition (EMT) related genes, and high infiltrating of M2 is associated with poor prognosis [9, 10]. In particular, the results of recurrent xenotransplantation ATRT mouse model suggest that M2 are also involved in the acquisition of chemoresistance [10]. The ways how M2 exert immunosuppression function can be summarized as follows. Firstly, M2 can express the death ligand Fas-L and bind to Fas receptor on immune cells to directly mediate their apoptosis [8]. Secondly, M2 express inhibitory ligands PD-L1/L2 and CD80/86, which bind to inhibitory receptors such as PD-1 and CTLA-4 constitutively expressed in immune cells to activate them, directly inhibiting TCR and BCR signals to restrain the antitumor function of T cells and B cells [11, 12]. Thirdly, M2 inhibits the function of T cells and NK cells by expressing non classical HLA molecules HLA-G or HLA-E, which connect the costimulatory signal molecule ILT2 of T cells and NKG2 of NK cells respectively [13]. In addition, M2 also inhibit T cells activity by the depletion of L-arginine that is required for T cells function in the TME through secreting arginase 1 (ARG1), an enzyme characteristically expressed in M2 to promote tumor growth and progression [14]. Many immunosuppressive cytokines and immune cells exert immunosuppressive effects partly through promoting M2 polarization. The interaction among them will be discussed later in detail.

As mentioned above, TAMs are pivotal to create immunosuppressive TME, and the crosstalk between macrophages and various immune cells and cytokines in TME plays an irreplaceable role. Notably, there are few reviews about interaction between macrophages and other cells in the TME, and most of them focused on elaborating a certain recognition mechanism. In this review, to form a comprehensive understanding of how macrophages mediate tumor immune escape, we summarize the main mechanisms of macrophages involved in tumor immune escape and related targeted therapies, which might lead to improved clinical protocols and potentially novel strategies for overcoming macrophage associated immune tolerance.

## Pathways involved in regulating the phagocytic signal of macrophages

### CD47/SIRPα

**Fig. 1:**
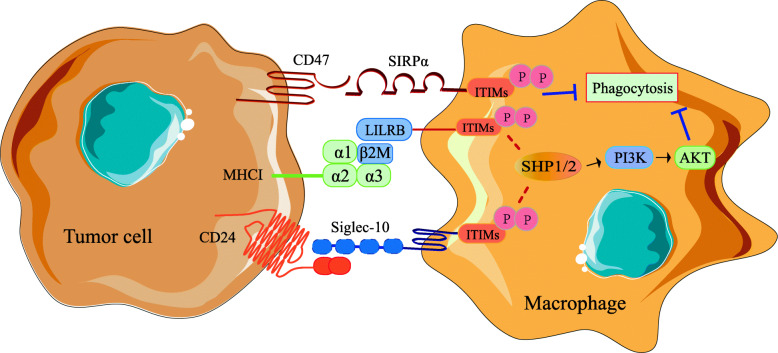
The mechanisms of macrophages participating in tumor antigen recognition disorder. Certain tumor cells highly express self-labels like CD47, β2M and CD24, which can phosphorylate ITIMs through CD47/SIRPα, β2M/LILRB and CD24/Siglec-10 signal axis respectively, activate downstream pathways, release anti phagocytic signal and negatively regulate phagocytic function of macrophages

CD47 is an immunoglobulin widely distributed on the surface of normal cells, which can negatively regulate anti-tumor immunity by inhibiting phagocytosis and participate in mediating cell proliferation, migration, apoptosis, and immune homeostasis [15]. Its main ligand, Signal-regulatory protein alpha (SIRPα) is a transmembrane protein highly expressed on the membrane of myeloid cells [16], and NH2-terminal in its extracellular region can bind to CD47, leading to tyrosine phosphorylation on immune receptor tyrosine-based inhibitory motifs (ITIMs), releasing a "don't eat me" signal, thereby inhibiting macrophage-mediated phagocytosis and protecting normal cells from destruction by the immune system [17] (Fig. 1). The universal expression of CD47 on the cell surface labels the cells as "self-labeling", by which macrophages distinguish "self" and "foreign" cells for phagocytosis [16].

Tumors cells highly express CD47 to avoid macrophage-mediated destruction. Studies have demonstrated that CD47 is highly expressed in a variety of tumors like hematological malignancy [18] and hepatocellular carcinoma (HCC) [19], by analyzing the clinical samples of tumor patients utilizing flow cytometry, western blot and immunohistochemistry, which is also associated with poor prognosis [20, 21]. A research of rhabdomyosarcoma indicated that after being co-cultured with tumor cells, the viability of macrophages dropped to 50–60%, which can be blocked by CD47 antibodies, implying that the immunosuppressive signal molecule CD47 allows cancer cells to escape from the elimination of macrophages innate immune response [22]. The above conclusions were further verified in the mouse model of small-cell lung cancer, figuring out that administration of CD47-blocking antibodies or targeted inactivation of the CD47 gene markedly inhibits tumor growth [23]. In addition, anti-CD47 therapy can also change the polarization state of macrophages in the TME. A study on glioblastoma found that CD47 blockade can enhance the phagocytic ability of macrophages and induce TAMs transform into an anti-tumor state [7].

### LILRB1/MHCI

Leukocyte immunoglobulin-like receptor B (LILRB) is expressed on most immune cells, composed of extracellular Ig-like regions, transmembrane regions and intracellular regions containing ITIMs. It can mediate the negative regulation of immune cells activation after binding with its main ligand major histocompatibility complex I (MHCI), which is a complex formed by HLAα chain and β2-microglobulin (β2M). After being phosphorylated by Src family protein tyrosine kinases, pITIM can recruit phosphatase containing Srchomology2 (SH2) domain to activate PI3K/AKT pathway, thereby negatively regulating the function of immune cells [24]. The fact that certain tumor cells highly express β2M, which could bind to LILRB1 on macrophages to inhibit phagocytosis, leading to the loss of immune surveillance, suggests that β2M is another self-label expressed by tumor cells [25]. Based on various cancer cell lines with or without expression of MHCI and CD47 as well as NSG mouse model of liver cancer, Barkal et al. confirmed that MHCI and CD47 are independent yet cooperative anti-phagocytic signals, and interference with MHCI/LILRB1 can enhance the phagocytosis of macrophages to tumor cells both *in vitro* and *in vivo*, which makes the signal axis an important regulator of innate immune cell response [25].

Therefore, in patients with normal or high expression of MHCI on tumor cells, drugs targeting the MHCI/LILRB1 axis may promote anti-tumor immune responses and play a synergistic effect with drugs targeting CD47/ SIRPα axis. The study of Amira et al. proved that MHCI can cooperate with CD47 to promote tumor cells escaping from immune surveillance, while blocking them could sensitize tumors to macrophage attack and indirectly enhance the function of other immune cells [25]. Studying the immunosuppressive mechanism of the MHCI/LILRB1 signal axis will help to develop therapeutic methods to restore the function of macrophages and control MHCI signaling in tumors.

### CD24/Siglec-10

CD24, also called heat stable antigen, is a highly glycosylated surface protein anchored by glycosylphosphatidylinositol, which could interact with sialic acid-binding immunoglobulin-like lectin-10 (Siglec-10) to reduce the innate immune-mediated noxious inflammation caused by infection or liver damage [26]. RNA sequencing data from TCGA and flow cytometry data from clinical breast and ovarian cancer patients demonstrated that tumor cells highly express CD24, while TAMs highly express Siglec-10 [27]. After binding with CD24, ITIM of Siglec-10 could recruit and activate the tyrosine phosphatase SHP-1 or SHP-2 containing SH2 domain, thereby blocking the cytoskeleton rearrangement required for macrophage phagocytosis, triggering an inhibitory signal transduction cascade [28]. It has been found that M0 expresses low level of Siglec-10, while M2 polarized cytokines like IL-4, TGF-β and IL-10 could induce strong expression of Siglec-10, indicating that the expression of Siglec-10 may be related to TAM-specific gene expression program. Co-culture of either wild type or ΔCD24 breast cancer cells with M2 revealed that CD24 deletion alone is sufficient to potentiate phagocytosis, while interfering CD24 or Siglec-10 could significantly enhance the phagocytic function of macrophages on CD24^+^ tumors and restrain the growth of tumor. Furthermore, in cancers resistant to CD47 blockade, anti-CD24 mAb enhance the phagocytic ability of macrophages, indicating there is a synergistic effect between CD24 interference and anti-CD47/SIRPα treatment [27].

“Don't eat me” signals CD47, β2M, and CD24, all of which involve ITIM-based macrophage signaling, indicating a conservative mechanism that leads to the immune selection of the subset of macrophage-resistant cancer cells, which allows tumor cells to escape from surveillance and clearance of macrophages. Therefore, grasping the mechanism by which tumor cells express anti-phagocytic signals can better predict therapeutic effect. Targeted drugs related to the above pathways have been developed, among which Hu5F9-G4 and CC-90002 are humanized mAb targeting human CD47, which can selectively eliminate malignant cells expressing CD47, mainly inducing transient anemia and mild neutropenia with no other obvious adverse reactions or autoimmune diseases [29]. Whereas, due to the widespread expression of CD47 in normal tissue, future research should still aim to optimize the structure of anti-CD47 preparation. Similarly, the combination of anti-human SIRPα antibody KWAR23 and tumor opsonizing antibody rituximab can significantly enhance the anti-tumor activity of neutrophils and TAMs [30]. Drugs aforementioned have clear targets and less adverse reactions, providing a theoretical basis and promising prospects for clinical application.

## Macrophages are involved in mediating immune suppression

### Interaction between cytokines and macrophages

#### IL-1

**Fig. 2:**
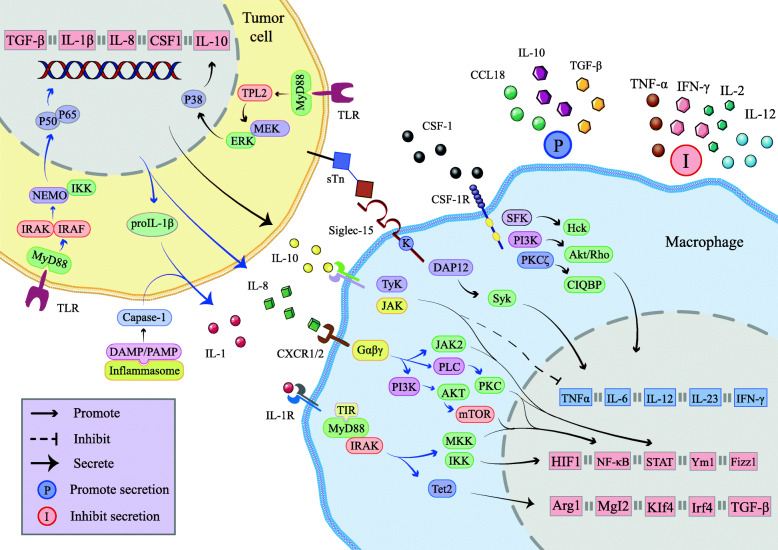
Secretory mechanism of major immunosuppressive cytokines in the TME. Tumor cells secrete IL-1, IL-8, IL-10, CSF-1 and other cytokines to bind to receptors on the surface of macrophages, regulating the expression of related immunosuppressive genes, increasing secretion of a variety of tumor-promoting factors such as TGF-β, inhibiting secretion of anti-tumor factors, so as to affect the immunity of main cells in the TME and promote tumor immune escape

IL-1 is an immunosuppressive cytokine with two different isoforms, IL-1α and IL-1β, which is mainly produced by tumor cells and immune regulatory cells through autocrine or paracrine in the TME and plays an important role in promoting tumor occurrence and development [31]. In tumor lesions, inflammasomes recognize pathogen-related molecular patterns or host-derived danger signal molecules to recruit and activate the pro-inflammatory protease caspase-1, which could cleave pro-IL-1β into bioactive IL-1β. Mechanistically, after IL-1α or IL-1β binds to IL-1R, the signaling adaptor MyD88 could be recruited and promote continuous nuclear factor Kappa-B (NF-κB) activation and the activity of mitogen-activated protein kinases (MAPK) through MyD88-IRAK signaling cascade [32] (Fig. 2). In the melanoma mouse model, ten-eleven-translocation-2 (Tet2) is found to be up-regulated on TAMs through IL-1R-MyD88 signaling. As a DNA methylcytosine dioxygenase, Tet2 is instrumental in DNA demethylation, by which maintains the expression of immunosuppressive genes comprising ARG1, MGL2, KLF4 and interferon regulatory factor 4 (IRF4) in TAMs and inhibits the antitumor function of tumor infiltrating T cells, resulting in significant tumor promotion in melanoma [33]. In addition, IL-1β induces the expression of CCL2 in TAMs and tumor cells, leading to the recruitment of myeloid cells such as myeloid derived suppressor cells (MDSCs) and TAMs into the TME [32]. Therefore, the way IL-1 promotes immunosuppression through TAMs should not be ignored.

Indeed, elevated IL-1 levels are positively correlated with tumor aggressiveness and poor prognosis in tumor models and cancer patients, indicating that targeting IL-1 is effective in anti-tumor therapy. Currently clinically available anti-IL-1 strategies encompass anti-IL-1α/IL-1β/IL-1Ra mAb, which have been shown to be well tolerated, reducing tumor cachexia and mortality in various clinical studies [31, 34]. A recent phase III clinical trial proved that antibodies targeting IL-1β significantly reduce the incidence and mortality of lung cancer [35]. The application of anti-IL-1α mAb MABp1 can greatly improve the survival of patients with advanced non-small cell lung cancer, ovarian cancer and other refractory cancers, without obvious side effects [34, 36]. IL-1 receptor antagonist Anakinra can block the induction of CCL22 by IL-1α, thereby reducing CCL22-mediated recruitment of TAMs and Tregs to the TME [37]. Antibody therapy targeting IL-1 is still under clinical research. Combining it with immune checkpoint inhibitors (ICIs) can activate antitumoral immune response more effectually, which is expected to become a new breakthrough for cancer immunotherapy.

#### IL-8

IL-8, also called CXCL8, is a pro-inflammatory chemokine whose main function is to induce the chemotaxis, infiltration and degranulation of neutrophils. Generally, chemotherapy or environmental stresses like hypoxia can promote TAMs to overexpress IL-8 and its receptors, while tumor cells could also be stimulated to secrete IL-8 after NF-κB is activated by TNF-α and IL-1α [38]. The G protein-coupled receptor undergoes conformational change after exposure to IL-8 and then couples with heterotrimeric G protein to activate PI3K or phospholipase C, leading to activation of AKT, PKC and MAPK signaling cascades, which upregulates the activity of a series of oncogene transcription factors comprising signal transducer and activator of transcription 3 (STAT3) phosphorylation [38]. In this way, major M2-related genes Ym-1 and Fizz-1 are significantly upregulated, promoting the expression of M2-related proteins such as CD204 and CD163, inducing the secretion of IL-10, vascular endothelial growth factor (VEGF) and monocyte chemoattractant protein-1 (MCP-1) that are beneficial to tumor growth, reducing the secretion of pro-inflammatory cytokines like IFN-γ, IL-12, IL-2 and MIP-1α, which has been observed in a mouse xenograft model with gastric cancer [39]. Therefore, carcinogenic pathway could be activated through IL-8 under abnormal conditions to promote tumor progression by mediating M2 polarization.

Among the blockers of IL-8-CXCR1/2 signaling pathway, the drugs targeting CXCR1/2 are more effective compared with the one targeting IL-8, which can be attributed to the fact that the activation of CXCR1 or CXCR2 by IL-8 can be compensated by other chemokines [38]. At present, several inhibitors and monoclonal antibodies against IL-8-CXCR1/2 pathway are in different stages of clinical trials, mostly with good tolerance and certain anti-tumor activity [40, 41]. For example, treating ovarian cancer with IL-8 neutralizing antibodies or CXCR2 inhibitor SB22500 can significantly inhibit its recurrence and metastasis [42], while combining with checkpoint inhibitors would induce more effective anti-tumor immune response [43]. Combining CXCR1 antagonist or IL-8 antibody with cytotoxic chemotherapy reduces the percentage of stem cells in breast cancer and effectively overcome chemoresistance [44]. Utilizing IL-8 neutralizing antibody HuMax-IL8 in patients with advanced malignant solid tumors has been tested in phase I clinical trial (NCT02536469), [45], and the CXCR1/2 antagonist Reparixin combined with Paclitaxel in patients with metastatic triple negative breast cancer has passed Phase I clinical trial [40], and its Phase II clinical trial has been launched (NCT02370238).

#### IL-10

IL-10 is an anti-inflammatory cytokine with a dual effect on tumor progression that depends on the specific tissue environment to exert anti-tumor immune response or promote tumor immune escape [46]. Accumulating studies have demonstrated that IL-10 can polarize TAMs towards the immunosuppressive M2 phenotype, which in turn secreted more IL-10 [47]. IL-10 receptor (IL-10R) contains two different receptor chains including IL-10R1 and IL-10R2. After IL-10R1 binds to IL-10, IL-10R2 acts as an auxiliary subunit to activate JAK1 and Tyk2, phosphorylate STAT3 and STAT1 [48]. STAT3 phosphorylation was reported to drive the production of BCL3, which plays a crucial role in regulating the dose-dependent effects of IL-10-induced suppression of M1-associated gene expression [49]. A recent study has suggested that microRNA let-7d inhibits M2 polarization of macrophages and subsequent tumor progression by decreasing mRNA expression levels of IL-10 in renal cell carcinoma (RCC) [50]. Another study in colorectal cancer also indicated that Wnt5a, mostly expressed in TAMs, activated CaKMII-ERK1/2-STAT3 pathway to induce macrophages to secrete IL-10 which then acted as an autocrine cytokine to induce M2 polarization [51]. Collectively, the immunosuppressive and tumor-promoting effect of IL-10 is related to M2 polarization of macrophages.

As an immunosuppressive cytokine, high serum concentration of IL-10 is associated with advanced stage and poor prognoses of cancer [52]. But IL-10 is also able to produce anti-tumor effects by inhibiting angiogenesis factors and improving the proliferation and cytotoxicity of CD8^+^T cells in the TME [53]. Studies have shown that increasing IL-10 serum concentration to a certain level can enhance the anti-tumor effect of CD8^+^ T cells, which has been verified in various solid tumors such as pancreatic cancer, lung cancer, and kidney cancer [54]. Nonetheless, the phase III clinical trial that assesses efficacy of IL-10R agonist Pegilodecakin combined with Oxaliplatin in patients with metastatic pancreatic cancer has ended in failure (NCT02923921). Therefore, further studies of IL-10-related cancer immunotherapy are still needed due to these complicated and contradictory biological effects.

#### M-CSF

In view of the important position of TAMs in TME, M-CSF, which can stimulate the proliferation and differentiation of monocytes, is also a fundamental cytokine [55]. M-CSF is a homodimeric glycoprotein existing in the form of secreted isoform (sM-CSF) and cell surface glycoproteins (mM-CSF), which can be expressed in common immune cells and tumor cells in TME [56]. At present, M-CSF existing in the circulation can promote M2 polarization is the most common cognition. After M-CSF binds to its receptor, downstream pathways PKC, PI3K and SFK could be activated to promote the migration of macrophages to tumor areas and transform them into M2 phenotype, and as mentioned earlier, they can also regulate the secretion of VEGF by macrophages and promote tumor angiogenesis [57, 58]. For example, lung cancer cells express Oct4 to up-regulate the secretion of M-CSF, promoting M2 polarization, leading to cancer growth and metastasis, which has been verified in syngeneic mouse lung tumor model and clinical samples of non-small cell lung cancer [59]. Analogously, based on athymic BALB/c mouse model and RCC cell line, researchers found that RCC cells co-expressed M-CSF and its receptor. In addition to recruiting and polarizing M2, they can also take advantage of M-CSF-mediated autocrine feedback loop aimed at promoting the repair of normal renal tubules, so as to trigger tumor cell proliferation and inhibit tumor cell apoptosis [60].

Whereas, it is worth noting that several early studies have described the antitumor effects of macrophages mediated by mM-CSF. Electron microscopic observation showed that tumorigenicity of glioma cells retrovirally transfected with mM-CSF gene were reduced and could be phagocytized by macrophages, suggesting the possible mechanism of mM-CSF-mediated cytotoxicity. Furthermore, the killing effect of macrophages on mM-CSF transfected clones could be blocked by a 100 folds excess of recombinant M-CSF, indicating that tumor cells transduced by mM-CSF are expected to become a safe live tumor cell vaccine [61, 62].

The difference of effect mediated by different isoforms of the same molecule may be explained by double signal model. When macrophages only receive sM-CSF activation signal, they would promote tumor growth and metastasis. However, when both signals are received, macrophages mediate tumor regression. Therefore, the balance of these two expressions form of M-CSF is of great significance. Clinical studies have proved that drugs targeting CSF-1R, such as Pexidartinib and PLX3397, are well tolerated in advanced solid tumors and have the potential to reduce TAMs infiltration [63–65].

#### TGF-β

TGF-β is produced by autocrine or paracrine from leukocyte lineage including lymphocytes and macrophages to manipulates their differentiation, proliferation, and state of activation. After siglec-15 expressed on TAMs is preferentially recognized by t tumor glycochain structural antigen sialyl-Tn, the secretion of TGF-β is promoted [66]. Similar to M-CSF, TGF also has dual effects. In the initial stage of carcinogenesis, TGF- β acts as the main tumor suppressor by applying cell inhibition and apoptosis procedures in tumor cells. Nevertheless, in the long-term pro-inflammatory environment, its original function is lost and reinterpreted to guide the tumor promoting function [67]. Study shows that siglec-15 is highly expressed in M2, and TGF-β can induce macrophages to polarize into the M2 phenotype, which also contributes to TGF-β secretion, thus forming a positive feedback loop [66]. After binding with TGF-βR, TGF-β inhibits activation and amplification of T cells as well as the secretion of IL-2, and further promote the differentiation of T cells into Th17 or Tregs, which has been identified in several cancer mice model [68–70]. Meanwhile, TGF-β prevents IFN from activating the phagocytosis phenotype of macrophages, but differentiated them into M2, thus preventing early immune activation and helping to build an immunosuppressive TME. Moreover, a study on xenograft nude mice model of liver cancer showed that M2 release TGF- β to mediate the binding of Smad2/3 to miR-362-3p promoter, resulting in the overexpression of miR-362-3p, which could reduce the maintenance of EMT by inducing the expression of CD82, so as to significantly promote the proliferation, invasion and metastasis of HCC cells [71]. Therefore, the role of TGF-β in TAMs-mediated immune escape can not be ignored.

Current anti-TGF-β treatment includes TGF-β or TGF-βR monoclonal antibodies [72, 73]. In several phase I/II clinical trials, TGF-βR1 inhibitors Galunisertib showed good safety and tolerability in the treatment of advanced solid tumor [74–76], and affect the survival of patients to some extent, but whether it can be used in clinical treatment requires further elucidation (Table 1).

**Table 1:** Representative clinical trials of macrophages-related key immunosuppressive cytokines

Target	NCT number	Drug name	Phase	Disease	Patients	Outcome	References
IL-1	NCT01327846	Canakinumab	III	Lung cancer	10061	The high-dose group reduced the risk of death by 77% (compared with placebo group)	[35]
IL-8	NCT02536469	BMS-986253	I	Advanced solid tumors	15	Safe, well tolerated	[41]
IL-8	NCT02583477	MEDI4736	II	Metastatic pancreatic ductal carcinoma	23	NA	-
CSF-1R	NCT01525602	PLX3397	Ib	Advanced solid tumors	54	Well tolerated, potential of reducing infiltration of TAMs	[64]
CSF-1R	NCT02371369	Pexidartinib	III	Tenosynovial giant cell tumour	174	Tolerable, overall response(39%)	[65]
TGF-β	NCT01582269	LY2157299	II	Recurrent glioblastoma	158	Failed to show improved overall survival	[75]
TGF-βR	NCT01246986	Galunisertib	II	Advanced hepatocellular carcinoma	149	Improved median survival rate of responders	[77]
PD-1	NCT02406781	Pembrolizumab	II	Sarcoma	57	The 6-month nonprogression rates were 0%, 0%, 14.3% for LMS, UPS and others respectively	[78]
PD-1	NCT02038946	Nivolumab	II	R/R follicular lymphoma	92	ORR was 4% and median PFS was 2.2 months	[79]

*ORR* Objective response rate, *PFS* progression-free survival, *OS* overall survival, *LMS* leiomyosarcoma, *UPS* undifferentiated pleomorphic sarcoma, *GIST* gastrointestinal stromal tumor

#### Exosomes

Exosomes are extracellular vesicles with a diameter of 30 to 150 nm, which contain substances such as proteins, nucleic acids and cytokines. Excluding direct contact between cells (short distance) and cytokines (long distance), exosomes are considered to be the third cell information exchange mechanism, and have been proved to act as a significant medium in the interaction between tumor cells and macrophages [80].

It has been proved in a variety of tumors that exosomes derived from tumor cells can promote the polarization of macrophages to M2. For example, in the *in vitro* co culture system of lung cancer cells and macrophages, tumor-derived exosomes enhance the oxygen consumption rate of macrophages and alter their bioenergetic state consistent with that of M2 macrophages after M0 internalized these exosomes [81]. Similar results have been verified in colorectal cancer, pancreatic cancer and other tumors [82–84]. In contrast, M2 derived exosomes (MDE) also affect invasion, drug resistance and immune escape of tumor cells. A representative example is that a study on colon cancer cell lines and BALB/C nude mouse model shows that, miR-21-5p and miR-155-5p are transferred to colorectal cancer cells through MDE and combined with BRG1 coding sequence to regulate the expression of BRG1, so as to mediate the migration and invasion of colorectal cancer cells and inhibit antitumor immune response [85]. Analogously, it has been found that exosomes-mediated transfer of functional CD11b/CD18 protein from TAMs to tumor cells may have the potential to enhance the migration potential of HCC cells [86]. In addition, researchers have found that M2 inhibit tumor cell apoptosis by down-regulating PTEN through miR-21 in exosomes, promoting cisplatin resistance in gastric cancer cells, which has been verified in athymic nude subcutaneous transplantation mice model [87]. Therefore, tumor or M2 related exosomes are potential therapeutic targets, which can be used to inhibit tumor proliferation and metastasis.

In addition to mediating the interaction between tumor cells and macrophages, exosomes also play a role in the crosstalk between macrophages and T cells, dendritic cells (DCs) and other cells. Therefore, in-depth study of the mechanism and mode of exosomes in the interaction between tumor cells and many other cells will help us fully understand their important role in tumor occurrence and development, and provide theoretical guidance for the tumor treatment of exosomes. Some researchers have synthesized responsive exosomes nano-bioconjugates for cancer therapy, which coupled the azide-modified exosomes derived from M1 with dibenzocyclooctyne-modified antibodies of CD47 and SIRPα. The nano-bioconjugates can release the antibody in acidic TME and reprogram the M2 to M1, leading to abolished “don't eat me” signaling and improved phagocytosis of macrophages [88]. Most of the research on the use of exosomes in the treatment of tumors is still in the exploratory stage, which has great research value.

### Interaction between macrophages and other immune cells

#### Macrophages participate in the formation of immunosuppressive myeloid microenvironment with MDSCs, DCs and TANs

MDSCs are the precursor cells of bone marrow-derived DCs, macrophages and granulocytes, recruited to tumor foci by chemokines like CCL2 and CCL5 to perform tumor immunosuppressive function [77], together forming an immunosuppressive tumor myeloid microenvironment [89]. We next discuss about the potential signal pathways by which macrophages participate in the formation of the immunosuppressive TME from the following aspects.

**Fig. 3:**
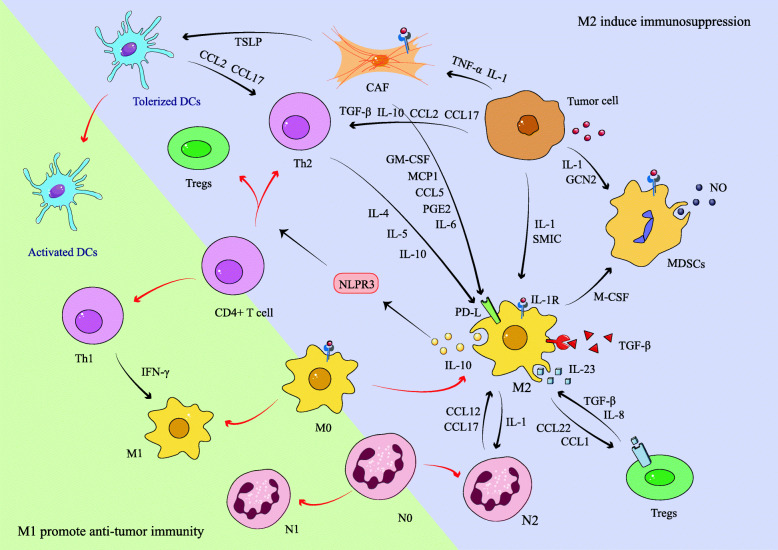
Crosstalk between major immunosuppressive cells in the TME promotes tumor immune escape. Tumor cells and immunosuppressive cells in the TME like Th2, Tregs and MDSCs can promote M0 polarize to M2 and enhance its proliferation and migration via a variety of signaling pathways. Conversely, M2 can also act on these cells, prompting them differentiate towards the immunosuppressive direction, thus forming an immunosuppressive the TME in concert. The red arrow indicates the polarization direction of cells, and the black arrow indicates that the cytokines can promote the polarization or function of corresponding cells

On the one hand, various mediators in the TME are involved in regulating the recruitment of MDSCs and monocytes as well as polarizing macrophages through different signaling pathways, thus promoting the formation of immunosuppressive myeloid microenvironment (Fig. 3). For example, General Control Nonderepressible 2 (GCN2) is required for tumor growth across several tumor types such as melanoma, colorectal tumor and lymphoma. The results of CyTOF and scRNA-seq of melanoma tumors present that the deletion of GCN2 alters the phenotype of macrophages and MDSCs by increasing the translation of CREB-2/ATF4, resulting in the loss of their inhibitory function and the enhancement of anti-tumor immunity of CD8^+^ T cell *in vivo* [90]. Another example is Nicotinamide phosphide bosyltransferase (NAMPT), which has been found to be a metabolic gate for mobilization of MDSC and could be motivated by CSF-1, inhibiting the transcription of CXCR4 through the NAD/Sirtuin1 axis driven by hypoxia-inducible factor 1 alpha (HIF1α), leading to MDSCs activation and M2 polarization in syngeneic orthotopic fibrosarcoma and mammary carcinoma mouse models [91]. Additionally, various cytokines are also involved in immunosuppressive function mediated by MDSCs and TAMs. IL-1 can recruit MDSCs and promote TAMs immunosuppressive programming in melanoma via the IL-1R-MYD88-Tet2 pathway to mediate immune escape [31]28813659. A recent study also indicates TAMs secrete TGF-β with positive feedback, and continued exposure to TGF-β and CSF-1 can promote the expansion of MDSCs and impair macrophages and DCs differentiation [92]. Moreover, flow cytometry analysis of TRAMP/MICB spontaneous prostate tumor model implies that the number of MDSCs in the spleen and tumor infiltration area significantly correlates with serum levels of soluble MHCI chain-related molecule (SMIC), the ligand of NKG2D, which activate STAT3 and induce MDSCs amplification as well as M2 polarization [93]. When recruited MDSCs migrate to the tumor region, hypoxia upregulates sialic acid transportation and combination to CD45, activating CD45 protein tyrosine phosphorases, resulting in rapid dephosphorylation and activity down-regulation of STAT3, which promotes the differentiation of MDSCs to TAMs in a non-HIF1-dependent manner [94]. Tumor tissues simultaneously up-regulate and down-regulate STAT3 activity in myeloid cells appear to be contradictory, but when time and space are taken into account, a dynamic hypothesis can be proposed. While MDSCs penetrate into tumor tissue from blood vessels, STAT3 could be up-regulated to amplify MDSCs by a mechanism mentioned above. After these recruited and amplified MDSCs further infiltrate tumor tissues and enter the deep vascular deficiency region, hypoxia-driven mechanism takes the lead to down-regulate STAT3 and promote the differentiation of MDSCs to TAMs. The two mechanisms switch dominance in different areas of tumor tissue, and promote the formation of immunosuppressive myeloid microenvironment sequentially and synergistically. Considering the irreplaceable role of MDSCs and macrophages in the immunosuppressive TME, many therapies such as Resveratrol, Toll-like receptor (TLR) 1/2 agonist, Gemcitabine nanoparticles, low-dose DNA methyltransferase and histone deacetylase inhibitors have been proposed to inhibit the recruitment and amplification of MDSCs and promote the polarization of M1 to break tumor immunosuppression [95–98].

**Fig. 4:**
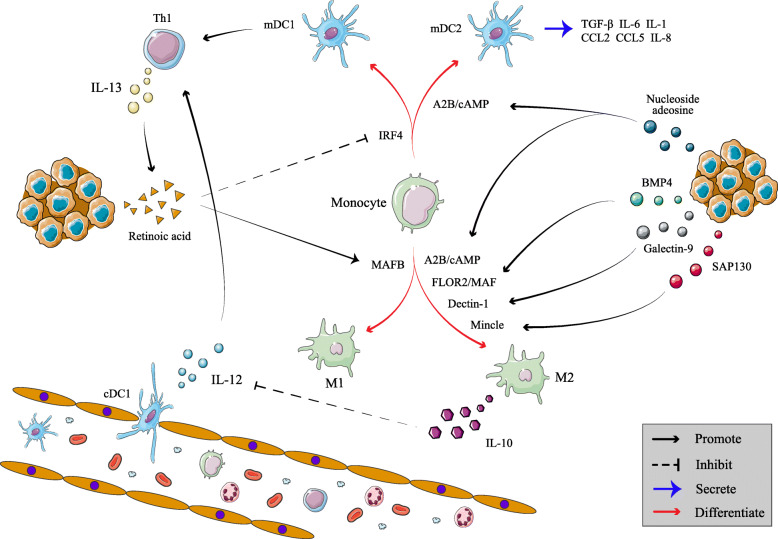
Multiple factors affect monocyte differentiation in the TME. High level of retinoic acid secreted by tumor cells can induce monocytes to differentiate into immunosuppressive macrophages by interfering with IRF4 and MAFB. Endogenous nucleoside adenosine induces abnormal development of DCs through A2B receptor, promoting activation of M2, synergistically enhancing immune escape. Similarly, other signaling molecules such as BMP4 and sap130 can also promote M2 polarization, and M2 secrete IL-10 to inhibit the secretion of IL-12 by vascular classic DCs (cDCs), thus inhibiting CD8^+^ T cell-dependent chemotherapy

Another noteworthy aspect is that different mediums related to tumor cells can regulate the differentiation of monocytes into DCs or inhibitory macrophages in TME. Previous studies have shown that IRF4 and musculoaponeurotic fibrosarcoma oncogene homolog B (MAFB) are critical in the differentiation of DCs and macrophages, respectively (Fig. 4). Tumor cells produce high levels of actinic acid promoted by IL-13 to inhibit IRF4, skewing monocytes differentiation toward TAMs rather than DCs in multiple murine sarcoma models [99]. According to earlier studies, tumor cells secret endogenous nucleoside adenosine to activate M2 and induce development of DCs through A2B receptor to promote immune escape. These “adenosine-induced” DCs fail to downregulate the expression of the monocytic marker CD14 but upregulate the DCs marker CD1a, impairing its ability to induce T cell proliferation [100]. Additionally, tumor cells express CTLRs’ dendritic cell-associated C-type lectins-1(Dectin-1) and macrophage-inducible C-type lectin (MINCLE) to combine with CTLRs expressed in myeloid cells, which inhibit activation of local myeloid cells and promote immune suppression [101]. *In vitro* experimental technologies like flow cytometry and ELISA were utilized to show that acute lymphocyte leukemia cells induce the production of immunosuppressive DCs and favour the generation of M2 by Bone Morphogenetic Protein 4 (BMP4) signal [102]. Thus, pharmacological blockade of these signaling pathways may be a therapeutic option for combating immune escape at the level of myeloid cell differentiation and polarization. In pancreatic ductal adenocarcinoma mice model, researchers used the small molecular agonists such as ADH-503 to partially activate CD11b, which is highly expressed on TAMs and tumor-infiltrating DCs, leading to immunosuppressive myeloid cells reprogramming, TAMs repolarization, DCs response enhancement and drug resistance overcoming in immunotherapy [103]. In subcutaneous tumor model of breast cancer, DMCA-pMIP-3β and FDMCA-pMIP-3β microsphere plasmid nanoparticles, an innovative targeting gene delivery system, have recently been proposed to promote the maturation of DCs and inhibit the M2 polarization by upregulating the macrophage protein 3 beta of tumor cells, which can also be a breakthrough direction [104].

On the other hand, researches on crosstalk among macrophages, MDSCs and DCs further verify the proposition mentioned above. Ovarian cancer cells highly express CD39 and CD73 that help to catalyze the conversion of extracellular ATP to adenosine, which can recruit monocytes and induce them to differentiate into IL-10-secreting TAMs. TAMs express CD39 and CD73 that further increase the infiltration of MDSCs and TAMs, thus forming a self-amplifying mechanism to promote local immune escape [105]. It has been found that MDSCs produces IL-10 to decrease the production of IL-12 and promote self-polarization to M2 in spontaneous metastatic 4T1 mouse mammary carcinoma, which requires intercellular contact and can be partially reversed by gemcitabine [106]. Meanwhile, macrophages are confirmed as the primary source of IL-10 which inhibits the secretion of IL-12 by TIDCs to weaken the efficacy of CD8^+^ T cell-dependent chemotherapy in untreated mammary carcinomas by evaluating FACS-sorted epithelial versus stromal cell populations [47].

Incidentally, tumor associated neutrophils (TANs) are also an important component of the immunosuppressive myeloid microenvironment. Like macrophages, neutrophils can be described as two subsets, N1 and N2, based on their different effects on immune cells in TME. N1 exhibit antitumor activity, either directly mediated by antibody-dependent cytotoxicity or indirectly mediated by the production of pro-inflammatory cytokines and T cells activation [107]. TANs, mostly N2, is considered to promote tumor metastasis [108]. Applying flow cytometry and immunofluorescence staining in Burkitt’s lymphoma Mice model identified that KWAR23 enhances antitumor activities of neutrophils and macrophages by binding SIRPα expressed on human myeloid cell subsets to block CD47/SIRPα discussed above [109]. In breast cancer models, TAMs secreted IL-1β induce γδ T cells release IL-17 to regulate the release of G-CSF and promote the recruitment of neutrophils to stimulate metastasis, indicating that TANs is closely related to TAMs in tumor progression [110]. However, the specific interaction mechanism between TANs and TAMs in tumorigenesis and immune escape remains under-revealed. Immunohistochemical results of the mouse model of combined injection of HCC cells and TANs indicate that TANs promote the intratumoral infiltration of TAMs and Tregs, which has been proved to be recruited through CCLl2/CCR2 and CCL17/CCR4 signaling pathways [111]. Although these results suggest the possibility that TANs interact with TAMs to promote immune escape during tumorigenesis, some findings seem to point in the opposite direction. Using a transgenic mouse model, researchers remove macrophages and neutrophils respectively during the occurrence of EGFR driven lung cancer, with the results that the removal of neutrophils has no effect on tumor formation, but the removal of macrophages significantly reduces the tumor load [112]. Similarly, using B11 to inhibit ephrinB2-EphB4 signaling in combination with radiation in preclinical models of pancreatic ductal adenocarcinoma would maximize the benefits of radiation therapy by significantly reducing Tregs, macrophages, and neutrophils infiltration and stromal fibrosis, leading to decreased tumor growth. Nevertheless, with or without radiation, the consumption of neutrophils alone is not sufficient to control tumor growth [113]. What is the reason for this divergence, the cancer type or the experimental design? Is it because TAMs-mediated immunosuppression possesses more compensatory mechanisms, so that the removal of TANs could not exert a substantive impact? The effect of this crosstalk on immune escape deserves further investigation.

In order to regulate immunosuppressive myeloid microenvironment, many other targeting methods have been proposed. As aforementioned, IL-10 is an important factor in immunosuppressive myeloid microenvironment. Delivering a synthetic microRNA mimic let-7b to activate TLR-7 and inhibit IL-10 production in breast cancer mouse model can effectively reprogram the function of TAMs/TIDCs, reverse the TME and inhibit tumor growth [114]. Because of the complex crosstalk between myeloid cells and other immune checkpoints [115], the mere consumption of TAMs may lead to compensatory recruitment of MDSCs [116] and up-regulation of ICIs [117], resulting in limitation of intratumoral myeloid targeting strategy. Therefore, the treatment of inhibitory microenvironment should be comprehensive. For instance, given that Notch signaling is associated with MDSCs, TAMs and Tregs infiltration as well as immune checkpoint upregulation, flow cytometry analysis of a Tgfbr1/Pten-knockout head and neck squamous cell carcinoma mouse model indicates that blocking Notch1 with γ-secretase inhibitor could reduce the impact of the above factors on TME to reduce tumor burden [118]. Furthermore, given that CD169^+^ macrophages rely on CD169 to transfer antigen to BATF3 signaling-dependent DCs to initiate CD8^+^ T cell immune response [119], delivering melanoma antigen to CD169 to induce DCs antigen presentation and trigger specific T cell responses, is an interesting attempt to systematically improve the defect of antigen presentation to circumvent the limitations of intratumoral myeloid targeting [120].

#### Interaction between macrophages and CD4^+^ T cells

Immature CD4^+^ T cells can be activated under various cytokine environments to differentiate into different T helper (Th) cell lines, including Th1, Th2, Th17 and Tregs, which play an important role in adaptive immunity [121]. A study utilizing scRNA-seq to analyze the tumor tissue of human advanced lung cancer before and after targeted therapies showed that T cells and macrophages as the main cell population presented a reversal of relative abundance in different stages of treatment. Compared with the samples in the stage of initial treatment or disease progression, the proportion of T cells in the immune cells of tumor samples in the remission state was larger, while the macrophages manifested an opposite infiltration pattern. Moreover, macrophages in the disease progression stage expressed high levels of IDO1 and chemokines like CXCL10 to recruit immunosuppressive cell populations such as Tregs and Th2 [122]. Based on this finding, we further explored the mutual regulation between macrophages and T cells. Tumor progression may be caused by Th1/Th2 mixed reaction or Th2 dominant response [123], relying on TGF-β and IL-10 to transform Th1 cells into Th2 cells to reverse the anti-tumor effects of CD8^+^ cytotoxic T cells and CD4^+^ Th1 cells, which is thought to be a tumor immune escape mechanism [124]. Th cells affect tumor TME by altering the polarization direction of macrophage. Th1 cells secrete IFN-γ to induce M1 polarization while Th2 cells could secrete IL-4, IL-5 and IL-10 to promote the generation of M2 macrophages [125]. Maria Pia Protti and Lucia De Monte sorted out the cytokine networks in pancreatic cancer: tumor cells release TNF-α and IL-1β to induce cancer associated fibroblasts (CAFs) to secrete thymic stromal lymphocyte (TSLP) to activate resident DCs, which can release Th2 chemokines (CCL2, CCL17) to recruit Th2 cells to the tumor region, and then Th2 cells could secrete Th2 cytokines to promote M2 polarization [126]. Relatively, macrophages also affect the differentiation direction of Th1/Th2. Studies have found that TAMs in pancreatic ductal adenocarcinoma could induce CD4^+^ T cells to differentiate into Th2 cells, Th17 cells and Tregs through NOD-like receptor family pyrin domain–containing 3 (NLRP3) signal relying on IL-10, meanwhile inhibit Th1 polarization and cytotoxic T cells activation [127]. A further supplementary demonstration of their interaction is based on the results of a study in BALB/c mice, suggesting Th2 upregulate PD-L2 on M2 via IL-4R α and STAT6, while Th1 upregulate PD-L1 expression on macrophages via TLR4 and STAT1 [128]. Merely blocking NLRP3 could prompt Th2 to repolarize to Th1, upregulating PD-L1 expression on macrophages, resulting in immune rebound, thus the combination of NLRP3 and PD-L1 targeting therapy may reverse the M2/Th2 immunosuppressive effect. Furthermore, tumor antigens in the TME may simultaneously activate extracellular signal-regulated kinase and p38 these two MAPKs, increasing IL-10 secretion, tilting Th1 response toward Th2 response, and consolidating M2/Th2-mediated immune escape [129]. Both of the mechanisms by which M2 induces Th2 polarization are IL-10 dependent, and IL-10, as mentioned above, also acts as a Th2 cytokine in M2 activation.

M2 and Th2-mediated immunosuppression have also been implicated in many clinical treatments. The presence of macrophages and Th2-polarized CD4^+^ T cells limits the efficacy of radiotherapy for breast cancer, and eliminating these cells or neutralizing IL-4 may improve the clinical response to cytotoxic therapy in breast cancer patients [130]. Analogously, in the radiotherapy of patent ductus arteriosus, flow cytometry shows that radiation exposure induce the infiltration of Th2 and M2, while M-CSF blocker could prevent the increase of them, so as to reduce the disease progression of radiotherapy-induced pre-invasive cancer and enhance the radiation antitumor effect [131]. Interestingly, single-cell analysis showed that anti-CD19-CAR-T cells did not polarize to Th1 or Th2, but showed a highly mixed Th1/Th2 response in the same cell population, with a small fraction of the cell population having Treg activity [132]. Whether the poor response to CAR-T therapy for solid tumors is associated with TAMs inducing this Th1/Th2 mixed functional state to tilt toward the Th2 dominant response, and whether the effect of macrophages on CAR-T cells is a potential mechanism of relapse after CAR-T therapy remains to be determined by more experiments.

Expressing Foxp3 as key transcription factor, CD4^+^ Tregs are mainly recruited from blood and acquire Treg phenotype and function after entering tumor tissue, exerting a significant action on immunosuppressive TME [133]. A recent study on murine as well as patients with non-small cell lung cancer reveals that Tregs secret IL-10 and IL-35 to promote exhaustion of CD8^+^ TILs to limit effective anti-tumor immunity, resulting in poor prognosis [134]. Macrophages could affect the proliferation, migration, and function of Tregs through various pathways. Firstly, flow cytometry analysis of glutamine-addicted RCC shows that TAMs induced by tumor-activated HIF1α could secrete IL-23, promoting the proliferation of Tregs and the expression of IL-10 and TGF-β, inhibiting the cytotoxic lymphocytes from killing tumor cells [135]. Studies on breast cancer using flow cytometric and RNA-seq analysis have also found that macrophages overexpress CCL1, the ligand of CCR8 expressed on Tregs, to attract Tregs to the tumor area [136]. Analogously, macrophages secrete CCL22 to induce Tregs to migrate into tumor region in ovarian cancer, inhibiting T cell immunity and promoting tumor growth [137]. Lung cancer researches shed more light on a self-amplifying immunosuppressive mechanism involve CCL22. TAMs-derived TGF-β can promote TAMs to secrete CCL22 and recruit Tregs, which could secrete high level of IL-8, further inducing TAMs to produce TGF-β and promote the formation of immunosuppressive microenvironment in malignant pleural effusion [138]. Relatively, Tregs are also involved in controlling macrophages recruitment, polarization and function, jointly maintaining the immunosuppressive TME. Tregs induce tumor-promoting activation of monocytes or macrophages through cell contact or secreting IL-10 [139], while flow cytometry analysis and immunohistochemistry of the MHCI-deficient mouse lymphoma demonstrated that Tregs deletion promote tumor cells to express MHCII and secrete cytokines and chemokines, enhancing anti-tumor activities [140]. Abundant targeting strategies for Tregs have been proposed, such as CCR4 monoclonal antibody and CTLA-4 antibody [141]. Naveen Sharma et. al treated a melanoma mouse model with intratumoral injection of TLR1/2 ligand Pam3CSK4 to increase Fcγ receptor IV expression on macrophages, leading to antibody-dependent macrophage-mediated depletion of Tregs and increasing efficacy of anti-CTLA-4 antibody in the combination treatment [142]. In addition, the combination of bromodomain inhibitor JQ1 and Ricolinostat can promote anti-tumor immunity in the mouse model of non-small cell lung cancer, the former specifically reduce the expression of Foxp3, CTLA-4 and PD-1 in Tregs and weaken its immunosuppressive function, while the latter could promote the expression of MHC and costimulatory molecule CD86 in macrophages [143].

#### Interaction between macrophages and CAFs

CAFs are the most abundant stromal cells in the TME, which can release a large number of cytokines, synthesize and remodel extracellular matrix to form tumor-promoting fibrous microenvironment [144]. 30% ~ 40% CAFs are generated from endothelial cells through endothelial-to-mesenmal transition, and could secrete HSP90α to induce M2 polarization and promote tumor progression [145], which can be abrogated by Octyl gallate by blocking HSP90Α-TLR4 ligation [146]. TAMs from peripheral blood mononuclear cell can enhance the proliferation of bone marrow mesenchymal stem cell (BM-MSCs), and CAFs from BM-MSCs can enhance the invasion of tumor and form a favorable environment for neuroblastoma [147]. Also, IL-6, M-CSF, MCP-1 and stromal cell-derived factor-1 can be secreted by CAFs to promote macrophages infiltration and differentiation [148], while M2 could relatively secrete TGF-β to promote endothelial-to-mesenmal transition and increase reactivity of CAFs, thus enhancing invasiveness of cancer cells [149]. In addition to the above targeting strategies, Cellax-DTX polymers can target αSMA^+^ CAFs and macrophages, continuously interact with them and consume them, thus preventing the development and metastasis of tumor cells, which has been applied in a metastatic PAN02 mouse model of pancreatic cancer [150]. In high-risk metastatic neuroblastoma model treated with a small molecule inhibitors targeted at microsomal prostaglandin E synthesis-1 (mPGES-1), immunohistochemical staining revealed a decrease in CD206 positive tumor-promoting M2 macrophages in tumors by blocking CAF-derived PGE2, which avoids adverse effect of current clinical PGE2-targeting drugs to reduces tumor growth [151]. The fact that macrophages and CAFs promote each other to enhance the growth of tumor makes the tumor immunotherapy complex and attractive.

To sum up, targeted blocking crosstalk between TAMs and various cells in the TME is a hotspot in current cancer immunotherapy, but related researches on the crosstalk between macrophages and immunosuppressive cells are still in the preclinical stage (Table 2). Further studies are needed to elucidate the potential mechanism of crosstalk between macrophages and immunosuppressive cells in tumor immunosuppression in order to develop effective target treatment strategies.

**Table 2:** Representative preclinical experiments about immunosuppressive cells

Cell	Target	Drug name	Animal model	Combination therapy	Effect on the TME	References
MDSC	NAMPT	FK866 and MV87	Fibrosarcoma mouse model	PD-1 antibody	Decrease MDSCs, increase T cell	[91]
	—	Resveratrol	Lewis lung carcinoma bearing mice	-	Decrease MDSCs, increase T cell	[95]
	γ-secretase	DAPT	Head and neck squamous cell carcinoma	-	Decrease MDSCs/TAM/Tregs	[118]
DCs	CD-11b	ADH-503	Pancreatic ductal adenocarcinoma	PD-1/41BB antibody	Promote M1 polarization; Increase cDCs/T cell	[103]
	TLR-7 /IL-10	let7b	Breast cancer	-	Activate TIDCs; Increase secretion of IL-12/IL-10	[114]
	CD169	CD169 antibodies	Melanoma	-	Increase CD8+T cell response	-
TANs	SIRPα	KWAR23	Burkitt’s lymphoma mice model	Anti-CD70 antibody	Enhance neutrophils and macrophages antitumor activity	[109]
	B11	EFNB2-EphB4	Pancreatic ductal adenocarcinoma	Radiation	Decrease Tregs, macrophages, and neutrophils	[113]
Tregs	IL-23	Guselkumab	Renal cell carcinoma	-	Decrease Tregs; Decrease secretion of IL-10/TGF-β; Increase cytotoxicity of CD8+T cells	[135]
	TLR1/2	Pam3CSK4	B16/F10 melanoma model	CTLA-4 antibody	Increase M1/T cells; Decrease Tregs	[142]
CAFs	Bromodoma	JQ1/ricolinostat	Non-small cell lung cancer model	-	Increase T cell, decrease M2	-
	HSP90	Octyl gallate	Pancreatic ductal adenocarcinoma	-	Decrease M2	[146]
	mPGES-1	CIII	Neuroblastoma tumor	-	Decrease M2	[151]

### Macrophages work with PD-1/PD-L1 axis to mediate immune escape

PD-L1, also known as CD274 or B7-H1, is a key protein expressed by tumor cells for suppressive immune response, and its receptor PD-1 (also known as CD279) is constitutively expressed in immune cells as a safety mechanism for controlling immune response [152]. Researchers applied CyTOF and scRNA-seq to characterize the immune map in tumor tissues of patients with RCC, which showed that macrophages possessed direct immunosuppressive characteristics, including the up-regulation of PD-1 related genes CD273 and CD274, which are responsible for T cell depletion [153]. After binding to PD-L1, the tyrosine residues of PD-1 are phosphorylated and combine with protein tyrosine phosphatase to activate downstream PI3K/Akt and other pathways to conduct inhibitory signals to T cells [154]. ICIs blocking PD-1/PD-L1 axis have shown great potential, while a part of cancer patients are still unable to benefit from ICIs (Table 1), triggering the exploration of the potential complicated relationship between macrophages and PD-1/PD-L1 axis in the TME [78, 79].

TAMs regulate the expression of PD-L1 on tumor cells and PD-1 on CD8^+^ T cells. PD-L1 is highly expressed in a variety of tumor tissues, can be up-regulated by TAM-derived TNF-α, and is positively correlated with macrophage infiltration in tumor stroma [155]. In a mouse model of lung cancer, macrophage-derived IL-6, which is promoted by Rab37 in a GTPase-dependent manner, up-regulates STAT3-denpendent PD-1 on CD8^+^ T cells to elicit an immunosuppressive TME [156]. The expression of PD-L1 on TAMs is also affected by many factors in the TME. IL-27/STAT3 axis induces overexpression of PD-L1/2 in lymphoma infiltrating macrophages [157]. Progranulin could up-regulate PD-L1 on TAMs through STAT3 pathway to promote immune evasion of breast cancer [158]. Meanwhile, PD-1/PD-L1 axis exerts a profound effect on macrophage function. The increase expression of PD-1 on TAMs could inhibit its phagocytosis [159] and structurally act on mTOR pathway, negatively regulating proliferation and activation of macrophages [160]. Therefore, PD-1/PD-L1 blockade can also directly affect macrophages.

PD-1/PD-L1 blockade can promote the pro-inflammatory polarization of macrophages [161], strengthen the activity of effector T cells, and also cooperate with other immune checkpoint inhibitors to limit tumor spread [162]. Nevertheless, Fcγ receptors (FcγRs) on macrophages migrate PD-1 antibodies that are isomorphic to human IgG4 such as nivolimab and pembrolizumab from T cells to TAM, weaken the efficacy of PD-1/PD-L1 blockade, suggesting Fc modified IgG variants and inhibiting FcγRs binding may be a solution to resistance [163]. Recently, many other therapeutic strategies targeting TAMs have been confirmed to work synergistically with PD-1/PD-L1 blocking therapy, such as ASF1A inhibitor, anti-CD40 antibody, CCL2-CCR2 axis blocker, VEGFR1 inhibitor, BRD4 inhibitor and so on [164–167].

In addition to PD-1/PD-L1 axis, some experimental results suggest that TAMs may also work with other immune checkpoints such as CTLA/CD86 axis and TIM3/galectin-9 axis to induce immune escape, but definitive evidence is still lacking [168, 169]. The interaction between macrophages and these immune checkpoints in immune escape deserves further study, and the effectiveness of various combination therapies also needs to be further confirmed by clinical trials.

### Macrophages help to form immune unresponsive sites

Immune unresponsiveness is an important reason for tumor tissues to evade immune surveillance, and macrophages may participate in immune privilege in tumor tissues through the following mechanisms. Firstly, macrophages could form a physiological barrier by accumulating CAFs to the tumor area, which deposit fibrous collagen, hyaluronic acid, fibronectin and other substances and secrete lysyl oxidase to stimulate type I collagen cross-linking, thereby forming a physical barrier [170]. Secondly, similar to tumor cells, the induced TAMs can express Fas-L and release active soluble Fas-L to induce apoptosis of Fas^+^ lymphocyte [171], while CAFs can up-regulate Fas-L and PD-L2 through MHC-1 antigen cross-presentation and inhibit the activity of CD8^+^ T cells [170], thus forming a TME lacking lymphocyte infiltration. Furthermore, Nobutaka Kobayashi et al. have developed a HA synthase 2 gene conditional knockout mice to prove that hyaluronic acid secreted by tumor cells can bind to TLR4 on the surface of macrophages, induce TAMs to migrate to tumor-related areas [172], and inhibit the expression of C/EBPβ through miR935 to promote the differentiation of macrophages into M2 Macrophages [173], thereby promoting malignant tumor cells to avoid immune surveillance and “cool down” the immunoreaction.

## Conclusion

The review summarizes a variety of immunosuppressive cells and pathways in the TME, and focuses on their interaction with macrophages and specific mechanisms involved in tumor immune escape. On the basis of existing research, we discussed several main mechanisms of how macrophages participate in tumor antigen recognition disorders and their interaction with immunosuppressive cytokines and cells, highlighting the core position of macrophages in the inhibitory cytokine network and its influence on tumor immune escape. So far, cytokine network of macrophage mediated immune escape have been studied profoundly, and related targeted drugs have uncovered a synergistic anti-tumor immune response in clinical treatment. Various types of treatment methods, such as inhibiting the up-regulation of macrophage-related surface molecules and the secretion of cytokines, and reducing their tumor-promoting polarization, can effectively enhance the phagocytosis of macrophages in the TME, and then interact with other immune cells. All of the above demonstrate the participation of macrophages in tumor immune escape through various molecular mechanisms, which deserve more attention. Nonetheless, in view of the complexity of cells crosstalk in the TME, related researches are still in the preclinical research stage. Therefore, given that macrophages-targeted therapy is expected to be the next frontier in cancer immunotherapy, clarifying their interaction mode would help to form a more comprehensive understanding of immunosuppressive TME, avoiding immune rebound, reducing tumor immune evasion and promoting the development of related research.

With the development of precision medicine, tumor therapy has gradually turned to targeted therapy, and immunotherapy will gradually be incorporated into comprehensive cancer therapy. Targeted elimination of immunosuppressive elements in the TME to enhance tumor killing activity is necessary for successful cancer treatment. Considering that immunosuppressive factors exist in the TME from the earliest stage of tumor formation, applying targeted agents that control or eliminate immunosuppressive factors in cancer treatment will effectively reduce immune escape and is a promising research direction. An in-depth understanding of tumor immune evasion mechanisms will help formulate effective treatment strategies to benefit cancer patients.

## Data Availability

Not applicable.

## Abbreviations

TAMs: Tumor Associated Macrophages
TME: Tumor Microenvironment
Tregs: Regulatory T cells
GM-CSF: Granulocyte Macrophage Colony-Stimulating Factor
M1: Classically Activated Macrophages
M2: Alternatively Activated Macrophages
scRNA-seq: Single-cell RNA sequencing
ATRT: Atypical Teratoid/Rhabdoid Tumors
CyTOF: Time-of-Flight Mass Cytometry
EMT: Epithelial-Mesenchymal Transition
ARG1: Arginase 1
SIRPα: Signal-regulatory Protein Alpha
ITIMs: Immune Receptor Tyrosine Inhibitory Motifs
HCC: Hepatocellular carcinoma
LILRB: Leukocyte Immunoglobulin-like Receptor B
MHCI: Major Histocompatibility Complex I
β2M: Beta 2 Microglobulin
SH2: Srchomology2
Siglec-10: Sialic Acid-binding Immunoglobulin-like Lectin-10
MAPK: Mitogen-activated Protein Kinase
NF-κB: Nuclear Factor Kappa-B
Tet2: Ten-eleven-translocation-2
IRF4: Interferon Regulatory Factor 4
MDSCs: Myeloid Derived Suppressor cells
ICIs: Immune Checkpoint Inhibitors
STAT3: Signal Transducer and Activator of Transcription 3
VEGF: Vascular Endothelial Growth Factor
MCP-1: Monocyte Chemoattractant Protein 1
MDE: M2 derived exosomes
DCs: Dendritic Cells
GCN2: General Control Nonderepressible 2
NAMPT: Nicotinamide Phosphide Bosyltransferase
HIF1α: Hypoxia-inducible Factor 1 Alpha
SMIC: Soluble MHCI Chain-related Molecule
TLR: Toll-like Receptor
MAFB: Musculoaponeurotic Fibrosarcoma Oncogene Homolog B
Dectin-1: Dendritic cell-associated C-type Lectins-1
MINCLE: Macrophage-inducible C-type Lectin
BMP4: Bone Morphogenetic Protein 4
TANs: Tumor associated neutrophils
Th cells: T Helper cells
CAFs: Cancer Associated Fibroblasts
TSLP: Thymic Stromal Lymphocyte
NLRP3: NOD-like Receptor Family Pyrin Domain–containing 3
BM-MSCs: Bone Marrow Mesenchymal Stem cells
mPGES-1: Microsoft Prostaglandin E synthesis-1
FcγRs: Fcγ Receptors

## References

[1] Weber JS, Yang JC, Atkins MB, Disis ML (2015). Toxicities of Immunotherapy for the Practitioner. J Clin Oncol.

[2] Seliger B (2005). Strategies of tumor immune evasion. BioDrugs.

[3] Haniffa M, Bigley V, Collin M (2015). Human mononuclear phagocyte system reunited. Semin Cell Dev Biol.

[4] Wynn TA, Chawla A, Pollard JW (2013). Macrophage biology in development, homeostasis and disease. Nature..

[5] Shapouri-Moghaddam A, Mohammadian S, Vazini H, Taghadosi M, Esmaeili SA, Mardani F (2018). Macrophage plasticity, polarization, and function in health and disease. J Cell Physiol.

[6] Anderson NR, Minutolo NG, Gill S, Klichinsky M (2021). Macrophage-Based Approaches for Cancer Immunotherapy. Cancer Res.

[7] Zhang M, Hutter G, Kahn SA, Azad TD, Gholamin S, Xu CY (2016). Anti-CD47 Treatment Stimulates Phagocytosis of Glioblastoma by M1 and M2 Polarized Macrophages and Promotes M1 Polarized Macrophages In Vivo. PLoS One.

[8] Noy R, Pollard JW (2014). Tumor-associated macrophages: from mechanisms to therapy. Immunity..

[9] Chung W, Eum HH, Lee HO, Lee KM, Lee HB, Kim KT (2017). Single-cell RNA-seq enables comprehensive tumour and immune cell profiling in primary breast cancer. Nat Commun.

[10] Melcher V, Graf M, Interlandi M, Moreno N, de Faria FW, Kim SN (2020). Macrophage-tumor cell interaction promotes ATRT progression and chemoresistance. Acta Neuropathol.

[11] Bloch O, Crane CA, Kaur R, Safaee M, Rutkowski MJ, Parsa AT (2013). Gliomas promote immunosuppression through induction of B7-H1 expression in tumor-associated macrophages. Clin Cancer Res.

[12] Butte MJ, Keir ME, Phamduy TB, Sharpe AH, Freeman GJ (2007). Programmed death-1 ligand 1 interacts specifically with the B7-1 costimulatory molecule to inhibit T cell responses. Immunity..

[13] Borrego F, Ulbrecht M, Weiss EH, Coligan JE, Brooks AG (1998). Recognition of human histocompatibility leukocyte antigen (HLA)-E complexed with HLA class I signal sequence-derived peptides by CD94/NKG2 confers protection from natural killer cell-mediated lysis. J Exp Med.

[14] Rodriguez PC, Quiceno DG, Zabaleta J, Ortiz B, Zea AH, Piazuelo MB (2004). Arginase I production in the tumor microenvironment by mature myeloid cells inhibits T-cell receptor expression and antigen-specific T-cell responses. Cancer Res.

[15] Burugu S, Dancsok AR, Nielsen TO (2018). Emerging targets in cancer immunotherapy. Semin Cancer Biol.

[16] Zhang W, Huang Q, Xiao W, Zhao Y, Pi J, Xu H (2020). Advances in Anti-Tumor Treatments Targeting the CD47/SIRPalpha Axis. Front Immunol.

[17] Bian Z, Shi L, Guo YL, Lv Z, Tang C, Niu S (2016). Cd47-Sirpalpha interaction and IL-10 constrain inflammation-induced macrophage phagocytosis of healthy self-cells. Proc Natl Acad Sci U S A.

[18] Gholamin S, Mitra SS, Feroze AH, Liu J, Kahn SA, Zhang M, et al. Disrupting the CD47-SIRPalpha anti-phagocytic axis by a humanized anti-CD47 antibody is an efficacious treatment for malignant pediatric brain tumors. Sci Transl Med. 2017;9(381). 10.1126/scitranslmed.aaf2968. 10.1126/scitranslmed.aaf2968 28298418

[19] Xiao Z, Chung H, Banan B, Manning PT, Ott KC, Lin S (2015). Antibody mediated therapy targeting CD47 inhibits tumor progression of hepatocellular carcinoma. Cancer Lett.

[20] Barrera L, Montes-Servin E, Hernandez-Martinez JM, Garcia-Vicente MLA, Montes-Servin E, Herrera-Martinez M (2017). CD47 overexpression is associated with decreased neutrophil apoptosis/phagocytosis and poor prognosis in non-small-cell lung cancer patients. Br J Cancer.

[21] Yang K, Xu J, Liu Q, Li J, Xi Y (2019). Expression and significance of CD47, PD1 and PDL1 in T-cell acute lymphoblastic lymphoma/leukemia. Pathol Res Pract.

[22] Herrmann D, Seitz G, Fuchs J, Armeanu-Ebinger S (2012). Susceptibility of rhabdomyosarcoma cells to macrophage-mediated cytotoxicity. Oncoimmunology..

[23] Weiskopf K, Jahchan NS, Schnorr PJ, Cristea S, Ring AM, Maute RL (2016). CD47-blocking immunotherapies stimulate macrophage-mediated destruction of small-cell lung cancer. J Clin Investig.

[24] Kang X, Kim J, Deng M, John S, Chen H, Wu G (2016). Inhibitory leukocyte immunoglobulin-like receptors: Immune checkpoint proteins and tumor sustaining factors. Cell Cycle.

[25] Barkal AA, Weiskopf K, Kao KS, Gordon SR, Rosental B, Yiu YY (2018). Engagement of MHC class I by the inhibitory receptor LILRB1 suppresses macrophages and is a target of cancer immunotherapy. Nat Immunol.

[26] Chen GY, Tang J, Zheng P, Liu Y (2009). CD24 and Siglec-10 selectively repress tissue damage-induced immune responses. Science..

[27] Barkal AA, Brewer RE, Markovic M, Kowarsky M, Barkal SA, Zaro BW (2019). CD24 signalling through macrophage Siglec-10 is a target for cancer immunotherapy. Nature..

[28] Chen HM, van der Touw W, Wang YS, Kang K, Mai S, Zhang J (2018). Blocking immunoinhibitory receptor LILRB2 reprograms tumor-associated myeloid cells and promotes antitumor immunity. J Clin Invest.

[29] Advani R, Flinn I, Popplewell L, Forero A, Bartlett NL, Ghosh N (2018). CD47 Blockade by Hu5F9-G4 and Rituximab in Non-Hodgkin's Lymphoma. N Engl J Med.

[30] Johnson LDS, Banerjee S, Kruglov O, Viller NN, Horwitz SM, Lesokhin A (2019). Targeting CD47 in Sezary syndrome with SIRPalphaFc. Blood Adv.

[31] Mantovani A, Barajon I, Garlanda C (2018). IL-1 and IL-1 regulatory pathways in cancer progression and therapy. Immunol Rev.

[32] Bent R, Moll L, Grabbe S, Bros M. Interleukin-1 Beta-A Friend or Foe in Malignancies? Int J Mol Sci. 2018;19(8):2155. 10.3390/ijms19082155 PMC6121377 30042333

[33] Pan W, Zhu S, Qu K, Meeth K, Cheng J, He K (2017). The DNA Methylcytosine Dioxygenase Tet2 Sustains Immunosuppressive Function of Tumor-Infiltrating Myeloid Cells to Promote Melanoma Progression. Immunity..

[34] Hong DS, Hui D, Bruera E, Janku F, Naing A, Falchook GS (2014). MABp1, a first-in-class true human antibody targeting interleukin-1alpha in refractory cancers: an open-label, phase 1 dose-escalation and expansion study. Lancet Oncol.

[35] Ridker PM, MacFadyen JG, Thuren T, Everett BM, Libby P, Glynn RJ (2017). Effect of interleukin-1beta inhibition with canakinumab on incident lung cancer in patients with atherosclerosis: exploratory results from a randomised, double-blind, placebo-controlled trial. Lancet..

[36] Coleman KM, Gudjonsson JE, Stecher M (2015). Open-Label Trial of MABp1, a True Human Monoclonal Antibody Targeting Interleukin 1alpha, for the Treatment of Psoriasis. JAMA Dermatol.

[37] Cavalli G, Dinarello CA (2018). Anakinra Therapy for Non-cancer Inflammatory Diseases. Front Pharmacol.

[38] Alfaro C, Sanmamed MF, Rodriguez-Ruiz ME, Teijeira A, Onate C, Gonzalez A (2017). Interleukin-8 in cancer pathogenesis, treatment and follow-up. Cancer Treat Rev.

[39] Li W, Zhang X, Wu F, Zhou Y, Bao Z, Li H (2019). Gastric cancer-derived mesenchymal stromal cells trigger M2 macrophage polarization that promotes metastasis and EMT in gastric cancer. Cell Death Dis.

[40] Schott AF, Goldstein LJ, Cristofanilli M, Ruffini PA, McCanna S, Reuben JM (2017). Phase Ib Pilot Study to Evaluate Reparixin in Combination with Weekly Paclitaxel in Patients with HER-2-Negative Metastatic Breast Cancer. Clin Cancer Res.

[41] Bilusic M, Heery CR, Collins JM, Donahue RN, Palena C, Madan RA (2019). Phase I trial of HuMax-IL8 (BMS-986253), an anti-IL-8 monoclonal antibody, in patients with metastatic or unresectable solid tumors. J Immunother Cancer.

[42] Yung MM, Tang HW, Cai PC, Leung TH, Ngu SF, Chan KK (2018). GRO-alpha and IL-8 enhance ovarian cancer metastatic potential via the CXCR2-mediated TAK1/NFkappaB signaling cascade. Theranostics..

[43] Dolgin E (2016). BMS bets on targeting IL-8 to enhance cancer immunotherapies. Nat Biotechnol.

[44] Samanta D, Gilkes DM, Chaturvedi P, Xiang L, Semenza GL (2014). Hypoxia-inducible factors are required for chemotherapy resistance of breast cancer stem cells. Proc Natl Acad of Sci U S A.

[45] Vacchelli E, Aranda F, Bloy N, Buque A, Cremer I, Eggermont A (2016). Trial Watch-Immunostimulation with cytokines in cancer therapy. Oncoimmunology..

[46] Mannino MH, Zhu Z, Xiao H, Bai Q, Wakefield MR, Fang Y (2015). The paradoxical role of IL-10 in immunity and cancer. Cancer Lett.

[47] Ruffell B, Chang-Strachan D, Chan V, Rosenbusch A, Ho CM, Pryer N (2014). Macrophage IL-10 blocks CD8+ T cell-dependent responses to chemotherapy by suppressing IL-12 expression in intratumoral dendritic cells. Cancer Cell.

[48] Oft M (2014). IL-10: master switch from tumor-promoting inflammation to antitumor immunity. Cancer Immunol Res.

[49] Chuang Y, Hung ME, Cangelose BK, Leonard JN (2016). Regulation of the IL-10-driven macrophage phenotype under incoherent stimuli. Innate Immunity.

[50] Su B, Han H, Gong Y, Li X, Ji C, Yao J (2021). Let-7d inhibits intratumoral macrophage M2 polarization and subsequent tumor angiogenesis by targeting IL-13 and IL-10. Cancer Immunol Immunother.

[51] Liu Q, Yang C, Wang S, Shi D, Wei C, Song J (2020). Wnt5a-induced M2 polarization of tumor-associated macrophages via IL-10 promotes colorectal cancer progression. Cell Communication Signaling.

[52] Lippitz BE (2013). Cytokine patterns in patients with cancer: a systematic review. Lancet Oncol.

[53] Ouyang W, O'Garra A (2019). IL-10 Family Cytokines IL-10 and IL-22: from Basic Science to Clinical Translation. Immunity..

[54] Naing A, Infante JR, Papadopoulos KP, Chan IH, Shen C, Ratti NP (2018). PEGylated IL-10 (Pegilodecakin) Induces Systemic Immune Activation, CD8(+) T Cell Invigoration and Polyclonal T Cell Expansion in Cancer Patients. Cancer Cell.

[55] Pixley FJ, Stanley ER (2004). CSF-1 regulation of the wandering macrophage: complexity in action. Trends Cell Biol.

[56] Douglass TG, Driggers L, Zhang JG, Hoa N, Delgado C, Williams CC (2008). Macrophage colony stimulating factor: not just for macrophages anymore! A gateway into complex biologies. Int Immunopharmacol.

[57] Dwyer AR, Greenland EL, Pixley FJ. Promotion of Tumor Invasion by Tumor-Associated Macrophages: The Role of CSF-1-Activated Phosphatidylinositol 3 Kinase and Src Family Kinase Motility Signaling. Cancers. 2017;9(6):68. 10.3390/cancers9060068 PMC5483887 28629162

[58] Wang Y, Su J, Yuan B, Fu D, Niu Y, Yue D (2018). The role of C1QBP in CSF-1-dependent PKCzeta activation and macrophage migration. Exp Cell Res.

[59] Lu CS, Shiau AL, Su BH, Hsu TS, Wang CT, Su YC (2020). Oct4 promotes M2 macrophage polarization through upregulation of macrophage colony-stimulating factor in lung cancer. J Hematol Oncol.

[60] Menke J, Kriegsmann J, Schimanski CC, Schwartz MM, Schwarting A, Kelley VR (2012). Autocrine CSF-1 and CSF-1 receptor coexpression promotes renal cell carcinoma growth. Cancer Res.

[61] Jadus MR, Chen Y, Boldaji MT, Delgado C, Sanchez R, Douglass T (2003). Human U251MG glioma cells expressing the membrane form of macrophage colony-stimulating factor (mM-CSF) are killed by human monocytes in vitro and are rejected within immunodeficient mice via paraptosis that is associated with increased expression of three different heat shock proteins. Cancer Gene Ther.

[62] Jadus MR, Irwin MC, Irwin MR, Horansky RD, Sekhon S, Pepper KA (1996). Macrophages can recognize and kill tumor cells bearing the membrane isoform of macrophage colony-stimulating factor. Blood..

[63] Autio KA, Klebanoff CA, Schaer D, Kauh JSW, Slovin SF, Adamow M (2020). Immunomodulatory Activity of a Colony-stimulating Factor-1 Receptor Inhibitor in Patients with Advanced Refractory Breast or Prostate Cancer: A Phase I Study. Clin Cancer Res.

[64] Wesolowski R, Sharma N, Reebel L, Rodal MB, Peck A, West BL (2019). Phase Ib study of the combination of pexidartinib (PLX3397), a CSF-1R inhibitor, and paclitaxel in patients with advanced solid tumors. Ther Adv Med Oncol.

[65] Tap WD, Gelderblom H, Palmerini E, Desai J, Bauer S, Blay JY (2019). Pexidartinib versus placebo for advanced tenosynovial giant cell tumour (ENLIVEN): a randomised phase 3 trial. Lancet..

[66] Takamiya R, Ohtsubo K, Takamatsu S, Taniguchi N, Angata T (2013). The interaction between Siglec-15 and tumor-associated sialyl-Tn antigen enhances TGF-beta secretion from monocytes/macrophages through the DAP12-Syk pathway. Glycobiology..

[67] David CJ, Massagué J (2018). Contextual determinants of TGFβ action in development, immunity and cancer. Nat Rev Mol Cell Biol.

[68] Cheng ML, Chen HW, Tsai JP, Lee YP, Shih YC, Chang CM (2006). Clonal restriction of the expansion of antigen-specific CD8+ memory T cells by transforming growth factor-{beta}. J Leukoc Biol.

[69] Chalmin F, Mignot G, Bruchard M, Chevriaux A, Vegran F, Hichami A (2012). Stat3 and Gfi-1 transcription factors control Th17 cell immunosuppressive activity via the regulation of ectonucleotidase expression. Immunity..

[70] Chen W, Jin W, Hardegen N, Lei KJ, Li L, Marinos N (2003). Conversion of peripheral CD4+CD25- naive T cells to CD4+CD25+ regulatory T cells by TGF-beta induction of transcription factor Foxp3. J Exp Med.

[71] Zhang Q, Huang F, Yao Y, Wang J, Wei J, Wu Q (2019). Interaction of transforming growth factor-β-Smads/microRNA-362-3p/CD82 mediated by M2 macrophages promotes the process of epithelial-mesenchymal transition in hepatocellular carcinoma cells. Cancer Sci.

[72] den Hollander MW, Bensch F, Glaudemans AW, Oude Munnink TH, Enting RH, den Dunnen WF (2015). TGF-beta Antibody Uptake in Recurrent High-Grade Glioma Imaged with 89Zr-Fresolimumab PET. J Nucl Med.

[73] Faivre S, Santoro A, Kelley RK, Gane E, Costentin CE, Gueorguieva I (2019). Novel transforming growth factor beta receptor I kinase inhibitor galunisertib (LY2157299) in advanced hepatocellular carcinoma. Liver Int.

[74] Fujiwara Y, Nokihara H, Yamada Y, Yamamoto N, Sunami K, Utsumi H (2015). Phase 1 study of galunisertib, a TGF-beta receptor I kinase inhibitor, in Japanese patients with advanced solid tumors. Cancer Chemother Pharmacol.

[75] Brandes AA, Carpentier AF, Kesari S, Sepulveda-Sanchez JM, Wheeler HR, Chinot O (2016). A Phase II randomized study of galunisertib monotherapy or galunisertib plus lomustine compared with lomustine monotherapy in patients with recurrent glioblastoma. Neuro-Oncology.

[76] Staff PO (2020). Correction: Biomarkers and overall survival in patients with advanced hepatocellular carcinoma treated with TGF-betaRI inhibitor galunisertib. PLoS One.

[77] Kumar V, Patel S, Tcyganov E, Gabrilovich DI (2016). The Nature of Myeloid-Derived Suppressor Cells in the Tumor Microenvironment. Trends Immunol.

[78] Toulmonde M, Penel N, Adam J, Chevreau C, Blay JY, Le Cesne A (2018). Use of PD-1 Targeting, Macrophage Infiltration, and IDO Pathway Activation in Sarcomas: A Phase 2 Clinical Trial. JAMA Oncol.

[79] Armand P, Janssens A, Gritti G, Radford J, Timmerman J, Pinto A (2021). Efficacy and safety results from CheckMate 140, a phase 2 study of nivolumab for relapsed/refractory follicular lymphoma. Blood..

[80] Guo W, Li Y, Pang W, Shen H (2020). Exosomes: A Potential Therapeutic Tool Targeting Communications between Tumor Cells and Macrophages. Mol Ther.

[81] Pritchard A, Tousif S, Wang Y, Hough K, Khan S, Strenkowski J, et al. Lung Tumor Cell-Derived Exosomes Promote M2 Macrophage Polarization. Cells. 2020;9(5):1303. 10.3390/cells9051303 PMC7290460 32456301

[82] Frank AC, Ebersberger S, Fink AF, Lampe S, Weigert A, Schmid T (2019). Apoptotic tumor cell-derived microRNA-375 uses CD36 to alter the tumor-associated macrophage phenotype. Nat Commun.

[83] Wang X, Luo G, Zhang K, Cao J, Huang C, Jiang T (2018). Hypoxic Tumor-Derived Exosomal miR-301a Mediates M2 Macrophage Polarization via PTEN/PI3Kγ to Promote Pancreatic Cancer Metastasis. Cancer Res.

[84] Zhao S, Mi Y, Guan B, Zheng B, Wei P, Gu Y (2020). Tumor-derived exosomal miR-934 induces macrophage M2 polarization to promote liver metastasis of colorectal cancer. J Hematol Oncol.

[85] Lan J, Sun L, Xu F, Liu L, Hu F, Song D (2019). M2 Macrophage-Derived Exosomes Promote Cell Migration and Invasion in Colon Cancer. Cancer Res.

[86] Wu J, Gao W, Tang Q, Yu Y, You W, Wu Z (2021). M2 Macrophage-Derived Exosomes Facilitate HCC Metastasis by Transferring α(M) β (2) Integrin to Tumor Cells. Hepatology (Baltimore, Md).

[87] Zheng P, Chen L, Yuan X, Luo Q, Liu Y, Xie G (2017). Exosomal transfer of tumor-associated macrophage-derived miR-21 confers cisplatin resistance in gastric cancer cells. J Exp Clin Cancer Res.

[88] Nie W, Wu G, Zhang J, Huang LL, Ding J, Jiang A (2020). Responsive Exosome Nano-bioconjugates for Synergistic Cancer Therapy. Angew Chem Int Ed Eng.

[89] Tran Janco JM, Lamichhane P, Karyampudi L, Knutson KL (2015). Tumor-infiltrating dendritic cells in cancer pathogenesis. J Immunol.

[90] Halaby MJ, Hezaveh K, Lamorte S, Ciudad MT, Kloetgen A, MacLeod BL, et al. GCN2 drives macrophage and MDSC function and immunosuppression in the tumor microenvironment. Sci Immunol. 2019;4(42):eaax8189. 10.1126/sciimmunol.aax8189 PMC7201901 31836669

[91] Travelli C, Consonni FM, Sangaletti S, Storto M, Morlacchi S, Grolla AA (2019). Nicotinamide Phosphoribosyltransferase Acts as a Metabolic Gate for Mobilization of Myeloid-Derived Suppressor Cells. Cancer Res.

[92] Gonzalez-Junca A, Driscoll KE, Pellicciotta I, Du S, Lo CH, Roy R (2019). Autocrine TGFbeta Is a Survival Factor for Monocytes and Drives Immunosuppressive Lineage Commitment. Cancer Immunol Res.

[93] Xiao G, Wang X, Sheng J, Lu S, Yu X, Wu JD (2015). Soluble NKG2D ligand promotes MDSC expansion and skews macrophage to the alternatively activated phenotype. J Hematol Oncol.

[94] Kumar V, Cheng P, Condamine T, Mony S, Languino LR, McCaffrey JC (2016). CD45 Phosphatase Inhibits STAT3 Transcription Factor Activity in Myeloid Cells and Promotes Tumor-Associated Macrophage Differentiation. Immunity..

[95] Zhao Y, Shao Q, Zhu H, Xu H, Long W, Yu B (2018). Resveratrol ameliorates Lewis lung carcinoma-bearing mice development, decreases granulocytic myeloid-derived suppressor cell accumulation and impairs its suppressive ability. Cancer Sci.

[96] Deng Y, Yang J, Qian J, Liu R, Huang E, Wang Y (2019). TLR1/TLR2 signaling blocks the suppression of monocytic myeloid-derived suppressor cell by promoting its differentiation into M1-type macrophage. Mol Immunol.

[97] Zhang Y, Bush X, Yan B, Chen JA (2019). Gemcitabine nanoparticles promote antitumor immunity against melanoma. Biomaterials..

[98] Lu Z, Zou J, Li S, Topper MJ, Tao Y, Zhang H (2020). Epigenetic therapy inhibits metastases by disrupting premetastatic niches. Nature..

[99] Devalaraja S, Folkert IW, Natesan R, Alam MZ, Li M, To TKJ (2020). Tumor-Derived Retinoic Acid Regulates Intratumoral Monocyte Differentiation to Promote Immune Suppression. Cell..

[100] Hasko G, Pacher P (2012). Regulation of macrophage function by adenosine. Arterioscler Thromb Vasc Biol.

[101] Chiffoleau E (2018). C-Type Lectin-Like Receptors As Emerging Orchestrators of Sterile Inflammation Represent Potential Therapeutic Targets. Front Immunol.

[102] Valencia J, MF-S L, Fraile-Ramos A, Sacedon R, Jimenez E, Vicente A, et al. Acute Lymphoblastic Leukaemia Cells Impair Dendritic Cell and Macrophage Differentiation: Role of BMP4. Cells. 2019;8(7):722. 10.3390/cells8070722 PMC6679123 31337120

[103] Panni RZ, Herndon JM, Zuo C, Hegde S, Hogg GD, Knolhoff BL, et al. Agonism of CD11b reprograms innate immunity to sensitize pancreatic cancer to immunotherapies. Sci Transl Med. 2019;11(499):eaau9240. 10.1126/scitranslmed.aau9240 PMC7197026 31270275

[104] He Y, Wang M, Li X, Yu T, Gao X (2020). Targeted MIP-3beta plasmid nanoparticles induce dendritic cell maturation and inhibit M2 macrophage polarisation to suppress cancer growth. Biomaterials..

[105] Montalban Del Barrio I, Penski C, Schlahsa L, Stein RG, Diessner J, Wockel A (2016). Adenosine-generating ovarian cancer cells attract myeloid cells which differentiate into adenosine-generating tumor associated macrophages - a self-amplifying, CD39- and CD73-dependent mechanism for tumor immune escape. J Immunother Cancer.

[106] Sinha P, Clements VK, Bunt SK, Albelda SM, Ostrand-Rosenberg S (2007). Cross-talk between myeloid-derived suppressor cells and macrophages subverts tumor immunity toward a type 2 response. J Immunol.

[107] Jackaman C, Tomay F, Duong L, Abdol Razak NB, Pixley FJ, Metharom P (2017). Aging and cancer: The role of macrophages and neutrophils. Ageing Res Rev.

[108] Güç E, Pollard JW (2021). Redefining macrophage and neutrophil biology in the metastatic cascade. Immunity..

[109] Ring NG, Herndler-Brandstetter D, Weiskopf K, Shan L, Volkmer JP, George BM (2017). Anti-SIRPα antibody immunotherapy enhances neutrophil and macrophage antitumor activity. Proc Natl Acad Sci U S A.

[110] Kersten K, Coffelt SB, Hoogstraat M, Verstegen NJM, Vrijland K, Ciampricotti M (2017). Mammary tumor-derived CCL2 enhances pro-metastatic systemic inflammation through upregulation of IL1β in tumor-associated macrophages. Oncoimmunology..

[111] Zhou SL, Zhou ZJ, Hu ZQ, Huang XW, Wang Z, Chen EB (2016). Tumor-Associated Neutrophils Recruit Macrophages and T-Regulatory Cells to Promote Progression of Hepatocellular Carcinoma and Resistance to Sorafenib. Gastroenterology.

[112] Saxon JA, Sherrill TP, Polosukhin VV, Sai J, Zaynagetdinov R, McLoed AG (2016). Epithelial NF-κB signaling promotes EGFR-driven lung carcinogenesis via macrophage recruitment. Oncoimmunology..

[113] Lennon S, Oweida A, Milner D, Phan AV, Bhatia S, Van Court B (2019). Pancreatic Tumor Microenvironment Modulation by EphB4-ephrinB2 Inhibition and Radiation Combination. Clin Cancer Res.

[114] Huang Z, Gan J, Long Z, Guo G, Shi X, Wang C (2016). Targeted delivery of let-7b to reprogramme tumor-associated macrophages and tumor infiltrating dendritic cells for tumor rejection. Biomaterials..

[115] Koelblinger P, Emberger M, Drach M, Cheng PF, Lang R, Levesque MP (2019). Increased tumour cell PD-L1 expression, macrophage and dendritic cell infiltration characterise the tumour microenvironment of ulcerated primary melanomas. J Eur Acad Dermatol Venereol.

[116] Baert T, Vankerckhoven A, Riva M, Van Hoylandt A, Thirion G, Holger G (2019). Myeloid Derived Suppressor Cells: Key Drivers of Immunosuppression in Ovarian Cancer. Front Immunol.

[117] Yang M, Du W, Yi L, Wu S, He C, Zhai W (2020). Checkpoint molecules coordinately restrain hyperactivated effector T cells in the tumor microenvironment. Oncoimmunology..

[118] Mao L, Zhao ZL, Yu GT, Wu L, Deng WW, Li YC (2018). gamma-Secretase inhibitor reduces immunosuppressive cells and enhances tumour immunity in head and neck squamous cell carcinoma. Int J Cancer.

[119] van Dinther D, Veninga H, Iborra S, Borg EGF, Hoogterp L, Olesek K (2018). Functional CD169 on Macrophages Mediates Interaction with Dendritic Cells for CD8(+) T Cell Cross-Priming. Cell Rep.

[120] van Dinther D, Lopez Venegas M, Veninga H, Olesek K, Hoogterp L, Revet M, et al. Activation of CD8(+) T Cell Responses after Melanoma Antigen Targeting to CD169(+) Antigen Presenting Cells in Mice and Humans. Cancers. 2019;11(2):183. 10.3390/cancers11020183 PMC6406251 30764534

[121] Zhu J, Yamane H, Paul WE (2010). Differentiation of effector CD4 T cell populations (*). Annu Rev Immunol.

[122] Maynard A, McCoach CE, Rotow JK, Harris L, Haderk F, Kerr DL (2020). Therapy-Induced Evolution of Human Lung Cancer Revealed by Single-Cell RNA Sequencing. Cell.

[123] Hamilton DH, Bretscher PA (2008). Different immune correlates associated with tumor progression and regression: implications for prevention and treatment of cancer. Cancer Immunol Immunother.

[124] Vinay DS, Ryan EP, Pawelec G, Talib WH, Stagg J, Elkord E (2015). Immune evasion in cancer: Mechanistic basis and therapeutic strategies. Semin Cancer Biol.

[125] Mills CD, Kincaid K, Alt JM, Heilman MJ, Hill AM (2000). M-1/M-2 macrophages and the Th1/Th2 paradigm. J Immunol.

[126] Protti MP, De Monte L (2012). Cross-talk within the tumor microenvironment mediates Th2-type inflammation in pancreatic cancer. Oncoimmunology..

[127] Daley D, Mani VR, Mohan N, Akkad N, Pandian G, Savadkar S (2017). NLRP3 signaling drives macrophage-induced adaptive immune suppression in pancreatic carcinoma. J Exp Med.

[128] Loke P, Allison JP (2003). PD-L1 and PD-L2 are differentially regulated by Th1 and Th2 cells. Proc Natl Acad Sci U S A.

[129] Halstead SB, Mahalingam S, Marovich MA, Ubol S, Mosser DM (2010). Intrinsic antibody-dependent enhancement of microbial infection in macrophages: disease regulation by immune complexes. Lancet Infect Dis.

[130] Shiao SL, Ruffell B, DeNardo DG, Faddegon BA, Park CC, Coussens LM (2015). TH2-Polarized CD4(+) T Cells and Macrophages Limit Efficacy of Radiotherapy. Cancer Immunol Res.

[131] Seifert L, Werba G, Tiwari S, Giao Ly NN, Nguy S, Alothman S (2016). Radiation Therapy Induces Macrophages to Suppress T-Cell Responses Against Pancreatic Tumors in Mice. Gastroenterology..

[132] Xhangolli I, Dura B, Lee G, Kim D, Xiao Y, Fan R (2019). Single-cell Analysis of CAR-T Cell Activation Reveals A Mixed TH1/TH2 Response Independent of Differentiation. Genomics Proteomics Bioinformatics.

[133] Wing JB, Tanaka A, Sakaguchi S (2019). Human FOXP3(+) Regulatory T Cell Heterogeneity and Function in Autoimmunity and Cancer. Immunity..

[134] Sawant DV, Yano H, Chikina M, Zhang Q, Liao M, Liu C (2019). Adaptive plasticity of IL-10(+) and IL-35(+) Treg cells cooperatively promotes tumor T cell exhaustion. Nat Immunol.

[135] Fu Q, Xu L, Wang Y, Jiang Q, Liu Z, Zhang J (2019). Tumor-associated Macrophage-derived Interleukin-23 Interlinks Kidney Cancer Glutamine Addiction with Immune Evasion. Eur Urol.

[136] Plitas G, Konopacki C, Wu K, Bos PD, Morrow M, Putintseva EV (2016). Regulatory T Cells Exhibit Distinct Features in Human Breast Cancer. Immunity..

[137] Curiel TJ, Coukos G, Zou L, Alvarez X, Cheng P, Mottram P (2004). Specific recruitment of regulatory T cells in ovarian carcinoma fosters immune privilege and predicts reduced survival. Nat Med.

[138] Wang D, Yang L, Yue D, Cao L, Li L, Wang D (2019). Macrophage-derived CCL22 promotes an immunosuppressive tumor microenvironment via IL-8 in malignant pleural effusion. Cancer Lett.

[139] Tiemessen MM, Jagger AL, Evans HG, van Herwijnen MJ, John S, Taams LS (2007). CD4+CD25+Foxp3+ regulatory T cells induce alternative activation of human monocytes/macrophages. Proc Natl Acad Sci U S A.

[140] Galani IE, Wendel M, Stojanovic A, Jesiak M, Muller MM, Schellack C (2010). Regulatory T cells control macrophage accumulation and activation in lymphoma. Int J Cancer.

[141] Tanaka A, Sakaguchi S (2017). Regulatory T cells in cancer immunotherapy. Cell Res.

[142] Sharma N, Vacher J, Allison JP (2019). TLR1/2 ligand enhances antitumor efficacy of CTLA-4 blockade by increasing intratumoral Treg depletion. Proc Natl Acad Sci U S A.

[143] Adeegbe DO, Liu Y, Lizotte PH, Kamihara Y, Aref AR, Almonte C (2017). Synergistic Immunostimulatory Effects and Therapeutic Benefit of Combined Histone Deacetylase and Bromodomain Inhibition in Non-Small Cell Lung Cancer. Cancer discovery.

[144] Chen X, Song E (2019). Turning foes to friends: targeting cancer-associated fibroblasts. Nat Rev Drug Discov.

[145] Fan CS, Chen LL, Hsu TA, Chen CC, Chua KV, Li CP (2019). Endothelial-mesenchymal transition harnesses HSP90alpha-secreting M2-macrophages to exacerbate pancreatic ductal adenocarcinoma. J Hematol Oncol.

[146] Chua KV, Fan CS, Chen CC, Chen LL, Hsieh SC, Huang TS. Octyl Gallate Induces Pancreatic Ductal Adenocarcinoma Cell Apoptosis and Suppresses Endothelial-Mesenchymal Transition-Promoted M2-Macrophages, HSP90alpha Secretion, and Tumor Growth. Cells. 2019;9(1):91. 10.3390/cells9010091 PMC7016987 31905895

[147] Hashimoto O, Yoshida M, Koma Y, Yanai T, Hasegawa D, Kosaka Y (2016). Collaboration of cancer-associated fibroblasts and tumour-associated macrophages for neuroblastoma development. J Pathol.

[148] Cho H, Seo Y, Loke KM, Kim SW, Oh SM, Kim JH (2018). Cancer-Stimulated CAFs Enhance Monocyte Differentiation and Protumoral TAM Activation via IL6 and GM-CSF Secretion. Clin Cancer Res.

[149] Comito G, Giannoni E, Segura CP, Barcellos-de-Souza P, Raspollini MR, Baroni G (2014). Cancer-associated fibroblasts and M2-polarized macrophages synergize during prostate carcinoma progression. Oncogene..

[150] Ernsting MJ, Hoang B, Lohse I, Undzys E, Cao P, Do T (2015). Targeting of metastasis-promoting tumor-associated fibroblasts and modulation of pancreatic tumor-associated stroma with a carboxymethylcellulose-docetaxel nanoparticle. J Control Release.

[151] Kock A, Larsson K, Bergqvist F, Eissler N, Elfman LHM, Raouf J (2018). Inhibition of Microsomal Prostaglandin E Synthase-1 in Cancer-Associated Fibroblasts Suppresses Neuroblastoma Tumor Growth. EBioMedicine..

[152] Sharpe AH, Pauken KE (2018). The diverse functions of the PD1 inhibitory pathway. Nat Rev Immunol.

[153] Chevrier S, Levine JH, Zanotelli VRT, Silina K, Schulz D, Bacac M (2017). An Immune Atlas of Clear Cell Renal Cell Carcinoma. Cell.

[154] Dong H, Strome SE, Salomao DR, Tamura H, Hirano F, Flies DB (2002). Tumor-associated B7-H1 promotes T-cell apoptosis: a potential mechanism of immune evasion. Nat Med.

[155] Tsukamoto M, Imai K, Ishimoto T, Komohara Y, Yamashita YI, Nakagawa S (2019). PD-L1 expression enhancement by infiltrating macrophage-derived tumor necrosis factor-alpha leads to poor pancreatic cancer prognosis. Cancer Sci.

[156] Kuo IY, Yang YE, Yang PS, Tsai YJ, Tzeng HT, Cheng HC (2021). Converged Rab37/IL-6 trafficking and STAT3/PD-1 transcription axes elicit an immunosuppressive lung tumor microenvironment. Theranostics..

[157] Horlad H, Ma C, Yano H, Pan C, Ohnishi K, Fujiwara Y (2016). An IL-27/Stat3 axis induces expression of programmed cell death 1 ligands (PD-L1/2) on infiltrating macrophages in lymphoma. Cancer Sci.

[158] Fang W, Zhou T, Shi H, Yao M, Zhang D, Qian H (2021). Progranulin induces immune escape in breast cancer via up-regulating PD-L1 expression on tumor-associated macrophages (TAMs) and promoting CD8(+) T cell exclusion. J Exp Clin Cancer Res.

[159] Gordon SR, Maute RL, Dulken BW, Hutter G, George BM, McCracken MN (2017). PD-1 expression by tumour-associated macrophages inhibits phagocytosis and tumour immunity. Nature..

[160] Hartley GP, Chow L, Ammons DT, Wheat WH, Dow SW (2018). Programmed Cell Death Ligand 1 (PD-L1) Signaling Regulates Macrophage Proliferation and Activation. Cancer Immunol Res.

[161] Xiong H, Mittman S, Rodriguez R, Moskalenko M, Pacheco-Sanchez P, Yang Y (2019). Anti-PD-L1 Treatment Results in Functional Remodeling of the Macrophage Compartment. Cancer Res.

[162] Fiegle E, Doleschel D, Koletnik S, Rix A, Weiskirchen R, Borkham-Kamphorst E (2019). Dual CTLA-4 and PD-L1 Blockade Inhibits Tumor Growth and Liver Metastasis in a Highly Aggressive Orthotopic Mouse Model of Colon Cancer. Neoplasia..

[163] Arlauckas SP, Garris CS, Kohler RH, Kitaoka M, Cuccarese MF, Yang KS, et al. In vivo imaging reveals a tumor-associated macrophage-mediated resistance pathway in anti-PD-1 therapy. Sci Transl Med. 2017;9(389):eaal3604. 10.1126/scitranslmed.aal3604 PMC5734617 28490665

[164] Li F, Huang Q, Luster TA, Hu H, Zhang H, Ng WL (2020). In Vivo Epigenetic CRISPR Screen Identifies Asf1a as an Immunotherapeutic Target in Kras-Mutant Lung Adenocarcinoma. Cancer Discov.

[165] Diggs LP, Ruf B, Ma C, Heinrich B, Cui L, Zhang Q, et al. CD40-mediated immune cell activation enhances response to anti-PD-1 in murine intrahepatic cholangiocarcinoma. J Hepatol. 2020;74(5):1145–54. 10.1016/j.jhep.2020.11.037 PMC9662232 33276030

[166] Yang H, Zhang Q, Xu M, Wang L, Chen X, Feng Y (2020). CCL2-CCR2 axis recruits tumor associated macrophages to induce immune evasion through PD-1 signaling in esophageal carcinogenesis. Mol Cancer.

[167] Lacal PM, Atzori MG, Ruffini F, Scimeca M, Bonanno E, Cicconi R (2020). Targeting the vascular endothelial growth factor receptor-1 by the monoclonal antibody D16F7 to increase the activity of immune checkpoint inhibitors against cutaneous melanoma. Pharmacol Res.

[168] Enninga EAL, Chatzopoulos K, Butterfield JT, Sutor SL, Leontovich AA, Nevala WK (2018). CD206-positive myeloid cells bind galectin-9 and promote a tumor-supportive microenvironment. J Pathol.

[169] Patel SS, Weirather JL, Lipschitz M, Lako A, Chen PH, Griffin GK (2019). The microenvironmental niche in classic Hodgkin lymphoma is enriched for CTLA-4-positive T cells that are PD-1-negative. Blood..

[170] De Jaeghere EA, Denys HG, De Wever O (2019). Fibroblasts Fuel Immune Escape in the Tumor Microenvironment. Trends Cancer.

[171] Brown SB, Savill J (1999). Phagocytosis triggers macrophage release of Fas ligand and induces apoptosis of bystander leukocytes. J Immunol.

[172] Kobayashi N, Miyoshi S, Mikami T, Koyama H, Kitazawa M, Takeoka M (2010). Hyaluronan deficiency in tumor stroma impairs macrophage trafficking and tumor neovascularization. Cancer Res.

[173] Zhang B, Du Y, He Y, Liu Y, Zhang G, Yang C (2019). INT-HA induces M2-like macrophage differentiation of human monocytes via TLR4-miR-935 pathway. Cancer Immunol Immunother.

